# Appraising the therapeutical potentials of *Alchornea laxiflora* (Benth*.*) Pax & K. Hoffm*.*, an underexplored medicinal herb: A systematic review

**DOI:** 10.3389/fphar.2022.958453

**Published:** 2022-12-02

**Authors:** Nem Kumar Jain, Mukul Tailang, Santosh Kumar, Balakumar Chandrasekaran, Yahia Alghazwani, Harish C. Chandramoorthy, Ashish Kumar, Hemali Deshpande, Pranay Wal, Manickam Balamurugan, Kumarappan Chidambaram

**Affiliations:** ^1^ School of Pharmacy, ITM University, Gwalior, Gwalior, Madhya Pradesh, India; ^2^ School of Studies in Pharmaceutical Sciences, Jiwaji University, Gwalior, Madhya Pradesh, India; ^3^ School of Sciences, ITM University, Gwalior, Gwalior, Madhya Pradesh, India; ^4^ Faculty of Pharmacy, Philadelphia University, Amman, Jordan; ^5^ Department of Pharmacology, College of Pharmacy, King Khalid University, Abha, Saudi Arabia; ^6^ Department of Microbiology and Clinical Parasitology, College of Medicine, King Khalid University, Abha, Saudi Arabia; ^7^ Center for Stem Cell Research, College of Medicine, King Khalid University, Abha, Saudi Arabia; ^8^ Department of Anatomy, College of Medicine, King Khalid University, Abha, Saudi Arabia; ^9^ Department of Pharmacy, Pranveer Singh Institute of Technology, Kanpur, India; ^10^ College of Pharmacy and Nursing, University of Nizwa, Nizwa, Oman

**Keywords:** *Alchornea laxiflora*, ethnopharmacology, African plants, traditional medicine, phytochemistry, pharmacology

## Abstract

**Ethnopharmacological relevance:** *Alchornea laxiflora* (Benth.) Pax & K. Hoffm. (Euphorbiaceae) is an important traditional medicinal plant grown in tropical Africa. The stem, leaves, and root have been widely used in the folk medicine systems in Nigeria, Cameroon, South Africa, and Ghana to treat various ailments, including inflammatory, infectious, and central nervous system disorders, such as anxiety and epilepsy.

**Material and methods:** The scientific name of the plant was validated using the “The Plant List,” “Kew Royal Botanic Gardens,” and Tropicos Nomenclatural databases. The literature search on *A. laxiflora* was performed using electronic search engines and databases such as Google scholar, ScienceDirect, PubMed, AJOL, Scopus, and Mendeley.

**Results:** To the best of our knowledge, no specific and detailed review has been reported on *A. laxiflora*. Consequently, this review provides an up-to-date systematic presentation on ethnobotany, phytoconstituents, pharmacological activities, and toxicity profiles of *A. laxiflora.* Phytochemical investigations disclosed the presence of important compounds, such as alkaloids, flavonoids, phenolics, terpenoids, and fatty acids. Furthermore, various pharmacological activities and traditional uses reported for this botanical drug were discussed comprehensively.

**Conclusion:** This systemic review presents the current status and perspectives of *A. laxiflora* as a potential therapeutic modality that would assist future researchers in exploring this African botanical drug as a source of novel drug candidates for varied diseases.

## Introduction

Indigenous herbal medicines are the first-line treatment for most third-world countries (Mahomoodally, 2013). According to the World Health Organization (WHO), about 80% of the world population employs herbal medicine for their primary health care using plant extracts (Mahomoodally, 2013; Nabatanzi et al., 2020). Various factors encourage herbal medicines, such as acceptability, poverty, cost-effectiveness, accessibility, and unavailability of modern health facilities (Hossain et al., 2014). However, there are concerns about the toxic effects of certain botanical drugs if used unchecked and irrationally (Firenzuoli and Gori, 2007; Okaiyeto and Oguntibeju, 2021). Globally, 28,187 plant species have been recorded as constituting medicinal use in 416 families of angiosperm plants. Euphorbiaceae is among the top three families with a significantly higher proportion of medicinal plants (Phumthum et al., 2019).

The Euphorbiaceae or spurge family comprises monoecious/dioecious herbs, shrubs, vines, or trees. Major groups of this family contain latex and have cosmopolitan distribution (Agbo et al., 2020). Economically important members include the natural rubber plant (*Hevea brasiliensis* (Willd. ex A. Juss.) Müll. Arg.), Tapioca plant (*Manihot esculenta* Crantz), castor oil (*Ricinus communis* L.), tung oil (*Vernicia fordii* (Hemsl.) Airy Shaw), candlenut oil (*Aleurites moluccanus* (L.) Willd.) and various oil, timber, medicinal, dye, and ornamental plants (Simpson, 2010; Agbo et al., 2020).

According to “The Plant List” (http://www.theplantlist.org), the *Alchornea* genus consists of 55 species. It has pan-tropical distribution with a strong tendency to tropical rain forests in American, African, and Asian countries. *Alchornea laxiflora* (Benth.) Pax & K. Hoffm. (*A. laxiflora*) is one of the accepted species of the *Alchornea* genus (Martínez et al., 2017).

**FIGURE 1:**
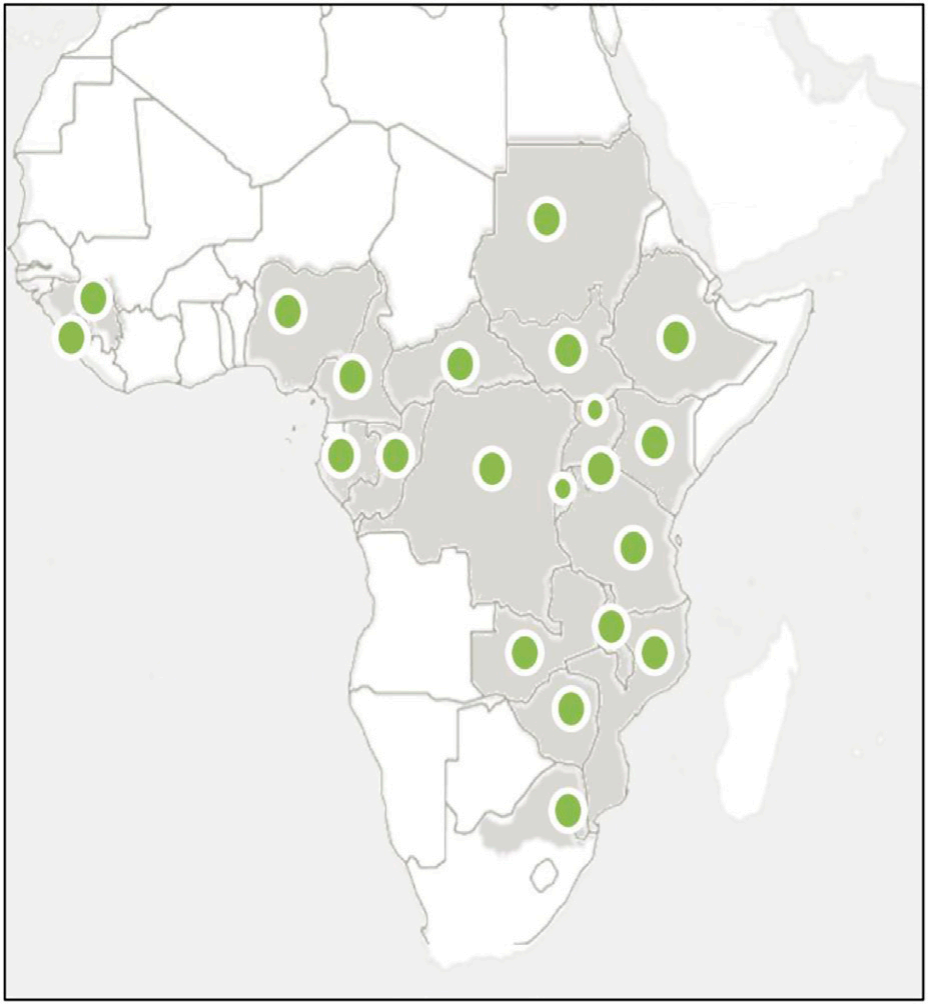
Geographical distribution of *A. laxiflora*, endemic to Africa.

*A. laxiflora* is endemic to Africa and is widely distributed (Figure 1) in central, eastern, and southern tropical African countries, namely, Burundi, Central African Republic, Congo, Ethiopia, Gabon, Guinea, Gulf of Guinea, Kenya, Malawi, Mozambique, Northern Provinces, Rwanda, Sierra Leone, Sudan, Swaziland, Tanzania, Uganda, Zambia, Zaïre, and Zimbabwe (Höft and Höft, 1995; Muller et al., 2005; Mwavu and Witkowski, 2008; Obodai and Nsor, 2009; Essiett and Ajibesin, 2010; Molander et al., 2014; McCarthy et al., 2017; Tegene, 2018; Verlhac et al., 2018; Magwede et al., 2019; Siwe-Noundou et al., 2019).

*A. laxiflora* grows best from the sea level up to 1,600 m altitude and is widely spread in evergreen forests, associated bushland in fire-protected places, and deciduous and riverine thickets near coasts (Burkill, 1985; Aweto, 2001; Obodai and Nsor, 2009). *A. laxiflora* primarily has four synonyms: *Alchornea engleri* Pax, *Alchornea schlechteri* Pax, *Lepidoturus laxiflorus* Benth., and *Macaranga thonneri* De Wild. The common names of *A. laxiflora* are “Lowveld bead string/Venda bead string/three veined bead string,” derived from the shape of its open inflorescences. It has several vernacular names depending on the cultural and ethnic diversity in Africa (Oladunmoye and Kehinde, 2011; Gbadamosi, 2015; Magwede et al., 2019). In the Ekiti state of Nigeria, it is also known as Canestiks and Arithmetic stick (Adeniran, 2015; Olanipekun and Aladetimiro (2017)). Some local African names are Uwenuwen, Ukpo-ubieka, Uwenriotan (Edo), Ububo (Igbo), Ijan, Ijun, Ijan furfur, Ijàndú, Igiiya, Pepe, Ewe lya, Ewe pepe, iyapepe, Opoto, Gbogbonse (Yoruba), Nwariwa (Ibibio), Urievwu, Urie vivu (Urhobo), Fura amarya (Hausa), Vendakralesnoer (Afrikaans), mubvamalofha and murundamalofha (Tshivenḓa), Murarahomba and Muruka (Shona), Eholo (Bakossi), Nnami (Kimwera), Mechango (Bambalang), Josos (Bakweri), Meshé (Bamoun), and Akwukwo Ugba (Njamen et al., 2013; Bafor et al., 2018, 2015; Akinpelu et al., 2015; Gbadamosi, 2015; Okokon et al., 2017b; Nwonu et al., 2018a; Bamimore and Elujoba, 2018; Magwede et al., 2019; Oluyemi and Blessing, 2019).

Recently, it has been established that medicinal plants are rich sources for new drug development, and traditional medicinal data has a quite good success rate in new therapeutics (Ochieng et al., 2022). Africa represents about a quarter of the world trade of biodiversity, and it is surprising that only a few drugs have been commercialized compared to other countries (Maroyi, 2016; Ali et al., 2017; Nabatanzi et al., 2020). The reason could be the lack of documentation, the secretive practices of local healers and folklore medicine practitioners, or lack of interest by first-world countries (Geethangili and Ding, 2018). *A. laxiflora* is one of the least explored plants possessing diverse ethnomedicinal and non-medicinal uses, as reported from different cultures and localities in Africa for centuries. However, it has gained the scientific interest of researchers in the last 2 decades regarding its pharmacological activities. A few studies have been conducted to identify and isolate the bio-constituents, and only limited reports are available for pharmacological studies. Although this plant has a wide distribution throughout Africa, it is worth noting that only Nigeria and Cameroon were the countries with the highest number of reports considering plant occurrence, traditional uses, and pharmacological activities.

**FIGURE 2:**
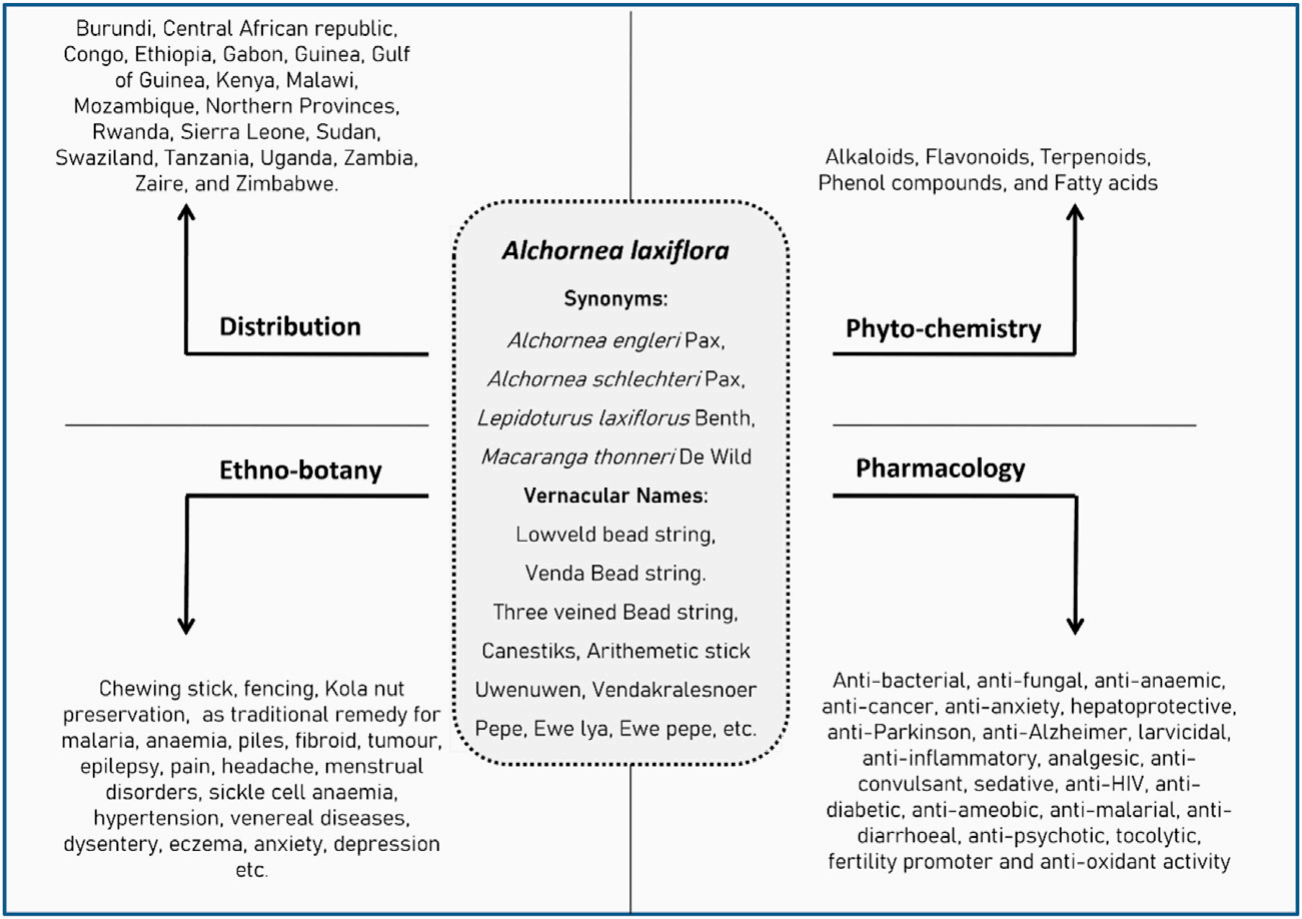
Summary of synonyms, geographical distribution, phytochemistry, ethnobotany, and pharmacology of *A. laxiflora*.

A couple of reviews broadly summarized the traditional and pharmacological uses of the *Alchornea* genus, primarily from *Alchornea cordifolia* (Schumach. & Thonn.) Müll. Arg. and *Alchornea floribunda* Müll. Arg. (Boniface et al., 2016; Agbo et al., 2020). However, no specific and detailed review has been reported in the literature on *A. laxiflora*. This review is intended to present detailed information systematically on the ethnobotany, phytochemistry, and pharmacology of *A. laxiflora*. Furthermore, this review will explore the therapeutic potential and evaluate future research opportunities pertaining to *A. laxiflora.* Figure 2 summarizes the crucial information on *A. laxiflora*.

## Methods

The literature search was done from various search engines and databases such as PubMed, Google Scholar, ScienceDirect, AJOL, Scopus, and Mendeley. We examined the literature published before June 2021 on ethnomedicinal uses, phytochemistry, pharmacology of extracts, and isolated compounds of *A. laxiflora*. Following the general guidelines on scientific nomenclature for plants to avoid any ambiguity and errors (Rivera et al., 2014), the species names, families, plant authority, and synonyms were verified using books, journal articles, and Webpages such as the “Kew Royal Botanic Gardens” (mpns.kew.org), the Missouri Botanical Garden’s Tropicos Nomenclatural database (www.tropicos.org), and “The Plant List” (www.theplantlist.org). The search terms “*Alchornea laxiflora*” or “*A. laxiflora* extract” or “*A. laxiflora* compounds” were used with no specified time limit. All articles with potential full-texts and titles/abstracts were included, and no language restrictions were applied. All the relevant references were checked for additional and unpublished citations. Most ethnobotanical data were collected from Nigeria, Cameroon, South Africa, and Zimbabwe. The pharmacological research literature on *A. laxiflora* was critically assessed for the general requirements of the pharmacological research of botanical drugs as suggested by Heinrich et al. (2020), and only the literature that met the general requirements was considered in this presented review.

## Botanical description and distribution

**FIGURE 3:**
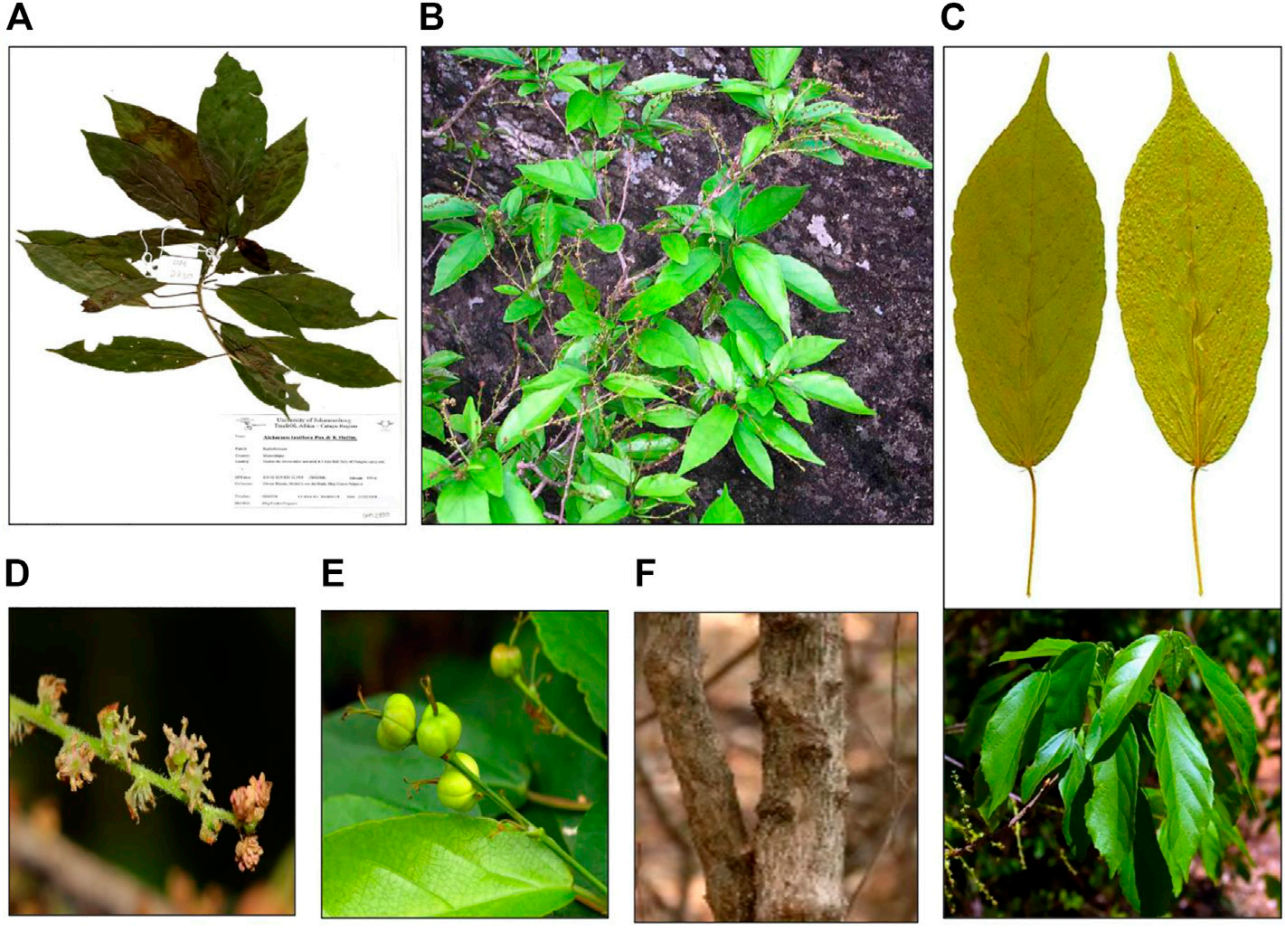
Morphology of *A. laxiflora*: herbarium specimen **(A)**, whole plant **(B)**, leaves **(C)**, inflorescence **(D)**, fruits **(E)**, and stem**(F)** (https://www.zimbabweflora.co.zw/).

*A. laxiflora* is a deciduous understorey tree or shrub that grows up to 6 m tall and is often found in places such as lowland tropical forests, wetlands, riverine vegetation, mixed deciduous woodlands, sub-montane forests, and semi-deciduous tropical rainforests (Mwavu and Witkowski, 2008; Obodai and Nsor, 2009; Tegene, 2018; Verlhac et al., 2018). The leaves are simple and alternate in arrangement, elliptic-lanceolate to oblong-lanceolate in shape, with dimensions of up to 17 × 8 cm. Moreover, the leaves are thinly structured, light green in color, turning to yellow, or red color in the dry season with three-veined venation from the base and shallowly crenate-serrate margination (Figure 3). The young leaves appear purple in color. The plant is monoecious, with male and female inflorescence on separate branches (Akinpelu et al., 2015). The flowers are unisexual on the same plant with conspicuous reddish bracts. The fruit is 5–7 mm in diameter with two-to-four-lobed capsules, which are thinly woody and blackish-brown (Hutchinson and Dalziel, 1937; Burkill, 1985). This plant has multiple traditional uses, but no attempts have been made to domesticate it. It was found to be key in re-sprouting woody species in natural or manmade disturbed forests (Mwavu and Witkowski, 2008). In Uganda, *A. laxiflora* was reported to be one of the several novel plant species used by Chimpanzees to make nests under anthropogenic pressure of habitat loss (McCarthy et al., 2017).

## Ethnobotany

Traditionally, all parts of *A. laxiflora* have utility in folk medicine for various purposes in different regions of Africa. Interestingly, maximum reports of traditional uses were from Nigeria and Cameroon, DR Congo, South Africa, and Ghana. However, no literature reports are noticed from Tanzania, Kenya, Kenya, Malawi, Mozambique, Northern Provinces, Rwanda, Sierra Leone, Sudan, Swaziland, Tanzania, Uganda, Zambia, Zaïre, and Zimbabwe. Other than medicinal uses, *A. laxiflora* has deep-rooted effects on the environmental and cultural aspects of Africa. A diversity of folk medicine applications of *A. laxiflora* are emmenagogue, promoting dental hygiene, easing toothache, and managing sickle cell diseases, as well as being anti-diabetic, anti-inflammatory, antioxidant, anti-infectious, anti-anemic, antifungal, and hepato-protective (Figure 2).

### Whole plant

In the South and Southwestern regions of Nigeria, this woody plant is an important component of rural architecture. Natives used to construct life fences (Fongod et al., 2014). In South Africa and DR Congo, *A. laxiflora* is reported to be used as an anti-venom for snake bites (Molander et al., 2014). Similarly, the whole plant has been reported for the treatment of malaria, pile, dysentery, eczema, cough, and high fever in the Ekiti state of Nigeria (Jayeoba et al., 2012; Adeniran, 2015).

### Leaves

Leaves are the most frequently used plant part in folk medicine preparations, taken orally in most instances as an infusion, decoction, or juice, followed by the stems, branches, and roots. Leaves crushed in water are sometimes applied externally to treat skin diseases (Ajibesin et al., 2007). Similarly, the leaves of *A. laxiflora* are squeezed and mixed with milk or cheese (wara). A cup of the resultant mixture is suggested to be taken twice a week as a remedy for anemia by the Yoruba tribe of southwest Nigeria (Fabeku and Akinsulire, 2008). For managing uterine contraction and prevention of miscarriages, traditional healers in the south of Nigeria use *A. laxiflora* leave concoction prepared by squeezing leaves in water, filtering them, and mixing them with calabash chalk or calabash clay, taken twice a day after conception for 6 months (Bafor et al., 2018). Moreover, in the food industry, *A. laxiflora* leaves are used as wraps to preserve Kola nuts (*Garcinia kola*) and other perishable food items (Ihejirika et al., 2015).

### Stem (bark and branchlets)

The stem branches are used as Chewing sticks (local toothbrushes) for cleaning teeth in Nigeria (Farombi et al., 2003). Stem bark and branches have also been used in traditional medicine for various purposes, notably for malaria, anemia, emmenagogue, ringworm, venereal disease, typhoid fever, antioxidant, infertility in females, infectious diseases, tumor, inflammation, teething problems, and toothache in South Africa, Ghana, and Nigeria (Dzoyem and Eloff, 2015; Kaur et al., (2012); Obodai and Nsor, 2009).

### Root

In the Ogun and Osun states of Nigeria, the roots and fruits are used as ethnomedicine to treat fibroids (Oyeyemi et al., 2019). In the Edo state of Nigeria, the root bark of *A. laxiflora* is boiled with an egg from local chicken and eaten as a remedy for hemorrhoids (Ugbogu and Chukwuma, 2019). The traditional practitioners in the Ekiti state of Nigeria also prescribe root decoction for lowering blood pressure and reducing incidences of heart failure (Adeniran and Falemu, 2017).

Traditional use of *A. laxiflora* is limited to Africa, with lots of similarities in therapeutic applications, possibly due to the shared cultural exchange of its ethnobotanical use. Table 1 shows the ethnomedicinal uses of *A. laxiflora* in different African countries, regions, and communities, together with the plant parts used.

**TABLE 1:** Traditional uses of *A. laxiflora* in different African countries.

Country	Region/community	Plant part/preparations	Traditional uses	References
Nigeria	Nigeria (general)	Leaves, stem, root	Malaria, anemia, emmenagogue, ringworm, venereal disease, typhoid fever, antioxidant	Ogundipe et al., 2001b; Farombi et al., 2003; Adeloye et al., 2005; Odugbemi and Akinsulire, 2008; Kaur et al., 2012
		Leaves decoction	Inflammatory and infectious diseases, administered to children with teething problems	
		Stem branches	Chewing stick (local toothbrush) for cleaning teeth, sexually transmitted diseases	
		Leaves	Antioxidant (preservation of kola nut and other perishable fruits and vegetables)	
	Yoruba tribe, southwest Nigeria	Leaves	Poliomyelitis, measles	Oladunmoye and Kehinde (2011)
	Southern Nigeria	Leave concoction	Uterine contraction/preterm labor prevention, sickle cell disorder	Amujoyegbe et al., 2016; Bafor et al., 2018
	Oyo state, Nigeria	Leaves squeezed in water	Sickle cell disease treatment	Gbadamosi (2015)
	Ekiti state, southwest Nigeria	Leaves	Menstrual disorder treatment (dysmenorrhea, oligomenorrhea, menorrhagia, amenorrhea)	Olanipekun and Aladetimiro (2017)
		Root decoction	Reduces incidences of heart failure, lowers blood pressure	Adeniran (2015)
		Whole plant, leaves, root, bark	Malaria, pile, dysentery, eczema, cough, and high fever	Jayeoba et al. (2012)
	Oyo state, Nigeria	Leaves	Systemic and nervous system infections	Borokini et al. (2013)
	Akwa Ibom state, Nigeria	Leaves crushed in water, applied externally	Skin diseases	Ajibesin et al. (2007)
	Oka Akoko, Ondo states, Nigeria	Leaves	Venereal diseases, promoting fertility	Olanipekun et al. (2016)
	Ondo state, Nigeria	Leaves infusion	Malaria treatment	Oyeyemi et al. (2019)
	Ogun and Osun states, Nigeria	Root and fruits	Fibroid treatment	Adebisi (2019)
	Badagry people of Lagos State	Leaves decoction	Diabetes	Makinde et al. (2015)
	Oyo, Ogun, and Osun states	Bark and fruits	Infertility in females	Soladoye et al. (2014)
	Imo state	Leaves	For wrapping Ugba (fermented African oil bean seeds)	Kabuo et al. (2013)
South Africa	—	Leaves, stem, branchlets	Infectious diseases, anti-tumor, inflammation, teething problems, chewing sticks	Dzoyem and Eloff (2015)
Ghana	—	Stem, branches	Treating toothaches	Obodai and Nsor (2009)
Cameroon	Bamun folk	Leaves	Urinary tract infections, hepatitis, pains, epilepsy, anxiety, insomnia, dizziness, headaches, and migraine	Njayou et al., 2008; Bum et al., 2009; Njamen et al., 2013; Fongod et al., 2014
	South and southwest region	Whole plant	Rural architecture, used to construct life fence		
	Ndop Central Sub-division	Leaves infusion	Postpartum pain	—	
	Bafia (Central Cameroon), Bazou and Foumbot (West Cameroon)	Leaves decoction	Stomachache, dysentery, jaundice, anxiety, depression	Ngnameko et al. (2019)	
DR Congo and South Africa	—	—	Snakebite treatment	Molander et al. (2014)	

## Phytochemistry

Although various phytochemical screening studies have suggested the presence of multiple classes of chemical constituents such as alkaloids, saponins, tannins, flavonoids, phenols, steroids, cardio-active glycosides, and reducing sugars, more studies are required to fully characterize the phytochemistry. However, the flowers and fruits of this plant species have not been studied extensively to identify phytoconstituents, relating to their low degree of non-usage in traditional medicines. The leaves, stems, and barks are highly exploited parts of the plant for the isolation and identification of phytoconstituents. The leaf has a higher diversity of phytochemicals compared to other plant parts. All the isolated compounds from *A. laxiflora* are mentioned in Table 2, and their chemical structures are presented in Supplementary Figures S1–S7.

**TABLE 2:** Phytoconstituents isolated from *A. laxiflora*.

Class of compound	Phytoconstituent	Code	Plant part	References
Flavonoids	Rutin	**1**	Leaves	Ogundipe et al. (2001a)
	Quercetin-3,4′-diacetate	**2**		
	Quercetin	**3**		Ogundipe et al., 2001b; Oloyede et al., 2011
	Quercetin-7,4′-disulphate	**4**		Ogundipe et al., 2001b; Oloyede et al., 2011
	Quercetin-3′,4′-disulphate	**5**		
	Quercetin-3,7,3′,4′-tetrasulphate	**6**		Oloyede et al. (2011)
	Quercetin-3-O-β-D-glucopyranoside	**7**		
	Quercitrin	**8**		Ogundipe et al., 2001a; Adeloye et al., 2005; Oloyede et al., 2011
	Taxifolin-3-O-β-D-galactopyranoside	**9**		Tapondjou et al. (2016)
	Taxifolin-3-O-β-D-xylopyranoside	**10**		
	Hyperoside	**11**		
	Reynoutrin	**12**		
	Guaijaverin	**13**		
Phenolic compounds	Phenol, 2,4-bis(1,1-dimethylethyl)-	**14**		Morah and Uduagwu (2017)
	Phthalic acid, butyl undecyl ester	**15**		
	Phenol, 2,6-bis(1,1-dimethylethyl) methyl-	**16**		
	Diisooctyl phthalate	**17**		
	Bis [di(trimethylsiloxy) phenylsiloxy] trimethylsiloxyphenylsiloxane	**18**		
	Syringaresinol-β-D-glucoside	**19**		Tapondjou et al. (2016)
	Ellagic acid	**20**	Stem bark	Sandjo et al. (2011)
	3-O-Methylellagic acid	**21**		
	3-O-Methylellagic acid-3-O-α-rhamnopyranoside	**22**		
	3,4,3′-tri-O-methylellagic acid	**23**		Mbaveng et al. (2015)
	Butylated hydroxyanisole	**24**	Leaves	Okokon et al. (2017b)
	Coniferyl alcohol	**25**		
	Pyrogallol	**26**		
	4-Vinylphenol	**27**		
	2-Methoxy-4-vinylphenol	**28**		
	Phthalic acid	**29**		
	1,1′ -Biphenyl-3,4,4′ -trimethoxy-6′ -formyl-	**30**	Root	Okokon et al. (2017a)
	3-Trimethylsilyloxyphenol	**31**		
	Zeranol	**32**		
Terpenoids	3-Acetyloleanolic acid	**33**	Stem bark	Sandjo et al. (2011)
	3-Acetoxyursolic acid	**34**		
	Adipedatol	**35**	Leaves	Bafor et al. (2018)
	Squalene	**36**	Root	Okokon et al. (2017a)
	Cholest-4-en-3-one	**37**		
	2,6,10-Trimethylundecan-(5*E*)-2,5,9-trien-4-one	**38**	Leaves	Okokon et al. (2017b)
	Isololiolide	**39**		
	β-Sitosterol-3-O-β-D-glucopyranoside	**40**	Stem bark, Leaves	Sandjo et al., 2011; Tapondjou et al., 2016
	Betulin	**41**	Leaves	Morah and Uduagwu (2017)
	2,2,4-Trimethyl-3-(3,8,12,16-tetramethyl-heptadeca3,7,11,15-tetraenyl)-cyclohexanol	**42**		
	Astaxanthin	**43**		
	Lycoxanthin	**44**		
	Rhodopin	**45**		
	Tetrahydrospirilloxanthin	**46**		
	Glycocholic acid	**47**		
	Ethyl iso-allocholate	**48**		
	Anhydrorhodovibrin	**49**		
	Hexahydrofarnesyl acetone	**50**		
	7,8-Epoxylanostan-11-ol, 3-acetoxy-	**51**		
	4-Vinylcholestan-3-ol	**52**		
	17-Hydroxyingenol	**53**		Bafor et al. (2018)
	Phytol	**54**		Okokon et al., 2017b; Morah and Uduagwu (2017)
Fatty acids	Palmitic acid	**55**		Otuechere et al. (2019)
	Oleic acid	**56**		
	Petroselinic acid	**57**		
	Ethyl oleate	**58**	Root	Okokon et al. (2017a)
	Methyl oleate	**59**		
	α-Linoleic acid	**60**		
	Propyl linoleate	**61**		
	Trimethylsilyl palmitate	**62**		
	Ethyl stearate	**63**		
	Ethyl tetracosanoate	**64**		
	11-cis-Octadecenoic acid trimethylsilyl ester	**65**		
	Elaidic acid trimethylsilyl	**66**		
	Pentadecanoic acid, ethyl ester	**67**		
	Ethyl laurate	**68**		
	Ethyl myristate	**69**		
	2*H*-Pyran-2-one, tetrahydro-4-hydroxy-6-pentyl-	**70**		
	Ethyl linoleate	**71**	Leaves	Morah and Uduagwu (2017)
	Icosyl oleate	**72**		
	Oleyl palmitoleate	**73**		
	Methyl isostearate	**74**		
	1-Heptatriacotanol	**75**		
	Cyclopropanedodecanoic acid	**76**		
	2-octyl-, methyl ester			
	Tricyclo [20.8.0.0 (7,16)] triacontane, 1(22),7(16)-diepoxy-	**77**		
	Ethanol, 2-(9,12-octadecadienyloxy)-, (Z, Z)-	**78**		
	9-Octadecene, 1,1′-[1,2-ethanediylbis (oxy)] bis-, (Z,Z)-	**79**		
	9-Desoxy-9x-chloroingol 3,7,8,12-tetraacetate	**80**		
	(10Z)-Tetradec-10-enoic acid-(2S)-2-carboxy-2-hydroxyethyl ester	**81**	Stem bark	Sandjo et al. (2011)
	(2R)-2-Hydroxy-N-[(2S,3S,4R,15Z)-1,3,4-trihydroxy-15-triaconten-2-yl] octacosamide	**82**		
	Pentadecanoic acid	**83**	Leaves	Okokon et al. (2017b)
	2-Hydroxyethyl oleate	**84**		
	Henicosyl formate	**85**		
	1,3-diacetyloxypropan-2-yl icosanoate	**86**		
	Methyl acetyl ricinoleate	**87**		
	Methyl linoleate	**88**		
	Methyl elaidolinolenate	**89**		
	1-Tetradecanol	**90**		
	1-Hexadecanol	**91**		
	Dimethyl undecanedioate	**92**		
	Z, E-2,13-octadecadien-1-ol	**93**		
	Methyl palmitate	**94**	Leaves, Root	Okokon et al., 2017a; Okokon et al., 2017b; Morah and Uduagwu (2017)
	Ethyl palmitate	**95**		Okokon et al., 2017a; Morah and Uduagwu, (2017)
	Stearic acid	**96**		Okokon et al., 2017a; Okokon et al., 2017b
	1-Heptacosanol	**97**		Okokon et al., 2017a; Morah and Uduagwu (2017)
Alkaloids	1,3,7,9-tetramethyluric acid	**98**	Leaves	Okokon et al. (2017b)
	4-Fluoro-2-nitroaniline, 5-[4-(pyrrolidin-1-yl) carbonylmethylpiperazin1-yl]-	**99**		Morah and Uduagwu (2017)
	Alchornealaxine	**100**		Tapondjou et al. (2016)
	Capsaicin	**101**	Root	Okokon et al. (2017a)
	Dihydrocapsaicin	**102**		
	Pheophorbide A	**103**	Leaves	Bafor et al. (2018)
Miscellaneous compounds	—	—	—	—
	Byzantionoside B	**104**	Leaves	Tapondjou et al. (2016)
	Leeaoside	**105**		
	2-Methylerythritol	**106**		Bafor et al. (2018)
	4-Amino-4-deoxyarabinose	**107**		
	3-Deoxy-arabino-hept-2-ulosonic acid	**108**		
	2-Amino-4,5-dihydroxy- 3,4-dimethylpentanoic acid	**109**		
	2-Methyl-3,5-dinitrobenzyl alcohol, tert-butyldimethylsilyl	**110**		Morah and Uduagwu (2017)
	Ether			
	3-Ethyl-5-(2-ethylbutyl)-octadecane	**111**		
	D-Galactitol, 3,6-anhydro-1,2,4,5-tetra-o-methyl-	**112**		Okokon et al. (2017b)
	10,11-Dihydro-10-hydroxy-2,3-dimethoxydibenz (b,f) oxepin	**113**		
	Hydroxy-4,4-dimethyldihydro-2(3H)-furanone	**114**		
	2-Coumaranone	**115**		
	2-Cyclopenten-1-one, 2-methyl-	**116**		
	(Z), (Z)-2,5-Dimethyl-2,4-hexadienedioic acid	**117**		
	1-Octadecene	**118**	Leave, Root	Okokon et al., 2017b; Morah and Uduagwu, 2017
	Propanoic acid, 3-(trimethylsilyl)-, ethyl ester	**119**	Root	Okokon et al. (2017a)
	2-Furancarboxylic acid, trimethylsilyl ester	**120**		
	Cyclopropenoic acid,1-trimethylsilyl, -2-(2-methylpropen-1-yl), methyl ester	**121**		
	2H-Pyran-2-one, 5,6-dihydro-6-pentyl-, (R)-	**122**		
	1,2,4-Cyclopentanetrione, 3-butyl-	**123**		
	1-Tetradecene	**124**		
	Octadecane, 1-bromo-	**125**		
	2-Butenoic acid, 2-methoxy-3-methyl-, methyl ester	**126**		
	Benzoic acid, 3-acetyloxy-, trimethylsilyl ester	**127**		
	Cyclopropanecarboxylic acid, 2,2-dimethyl-3-cis-(2-methyl-3-buten-2-yl)-	**128**		
	1-Hexadecene	**129**		
	Benzeneacetic acid, alpha-[(trimethylsilyl)oxy]-	**130**		
	2-Hydroxy-3-methoxybenzaldehyde, trimethylsilyl ether	**131**		
	Benzene, (2-ethyl-4-methyl-1,3-pentadienyl)-,* (E)-*	**132**		

Bold values are the numbers mentioned for Chemical structures in the supplementary file.

### Flavonoids

Flavonoids are the most common group of natural polyphenolic substances found in all fruits and vegetables. The flavonoids reported in *A. laxiflora* are in the form of flavanol and glycoside. From the ethyl acetate soluble fraction of the crude methanolic leaf extract of *A. laxiflora*, one novel acetylated flavonoid quercetin-3,4′-diacetate and three known flavonoid glycosides, quercetin, quercitrin, and rutin, were isolated (Ogundipe et al., 2001b). Concurrently, two novel sulfated flavonoids were isolated for the first time in the genus *Alchornea* and the family Euphorbiaceae, namely, quercetin-7,4′-disulphate and quercetin-3′,4′-disulphate (Ogundipe et al., 2001a). In another study, taxifolin glycosides were isolated from an *n*-butanol fraction of crude 50% ethanol aqueous leaf extract by AGC and SLHC chromatography characterized by spectroscopy techniques such as MS, ^1^H, and ^13^C NMR (Adeloye et al., 2005). Oloyede et al. (2011) reported that *A. laxiflora* leaves’ ethanol extract on fractionation and characterization yielded two new flavonoids, namely, quercetin-3-O-β-D-glucopyranoside and quercetin-3,7.3′,4′-tetrasulphate. Lately, five known flavonoid glycosides (hyperoside, reynoutrin, guaijaverin, taxifolin-3-O-β-D-xylopyranoside, taxifolin-3-O-β-D-galactopyranoside) and two megastigmane glycosides (byzantionoside B and leeaoside) together with one steroidal glycoside (β-sitosterol-β-D-glucoside) and one lignan glycoside (syringaresinol-β-D-glucoside) were isolated from the methanolic leaf extract of *A. laxiflora* (Tapondjou et al., 2016). Interestingly, flavonoids were reported mainly from the leaves, but other parts of the plant have not been investigated yet, and quercetin sounds to be the most abundant and common monomer in the plant (Table 2; Supplementary Figure S1).

### Phenolic compounds

The leaves of *A. laxiflora* have demonstrated a higher concentration of phenolic compounds than the other parts (Table 2). The phytochemical investigation of methanolic extract from the stem bark of *A. laxiflora* resulted in the isolation of eight compounds, including ellagic acid, 3-O-methylellagic acid, and 3-O-methylellagic acid-3-O-α-rhamnopyranoside (Sandjo et al., 2011). A novel ellagic acid derivative, namely, 3,4,3′-tri-O-methylellagic acid, was isolated from the methanolic extract obtained from the *A. laxiflora* stem bark (Mbaveng et al., 2015). In another study, Morah and Uduagwu (2017) separated several phenolic compounds, namely, phenol, 2,4-bis(1,1-dimethylethyl)-; phenol, 2,6-bis(1,1-dimethylethyl)-; phthalic acid, butyl undecyl ester; diisooctyl phthalate and bis[di(trimethylsiloxy) phenylsiloxy] trimethylsiloxy phenyl siloxane from the petroleum ether; and ethanol extract of *A. laxiflora* leaves. In addition, Okokon et al. (2017b) isolated butylated hydroxy anisole (BHA), pyrogallol, 1,2-benzenedicarboxylic acid; 4-((1*E*)-3-hydroxy-1-propenyl)-2-methoxyphenol; 2-methoxy-4-vinylphenol; and 4-vinylphenol as major constituents of *A. laxiflora* leaves. 1,1′-Biphenyl-3,4,4′-trimethoxy-6′-formyl-; phenol, 3-[(trimethylsilyl)oxy]-; and zeranol were reported to be isolated from the *A. laxiflora* root ethyl acetate fraction (Okokon et al., 2017a) (Table 2; Supplementary Figure S2).

### Terpenoids

Many terpenoids (22 compounds), including seven triterpenoids, two diterpenoids, one sesquiterpene, five carotenoids, and seven steroids, have been isolated from the leaves, stem bark, and root extracts of *A. laxiflora* (Table 2; Supplementary Figure S3). Triterpenoids are represented by four pentacyclic triterpenoids (3-acetyloleanolic acid, 3-acetoxyursolic acid, adipedatol, and betulin) and two squalene types (squalene and 2,2,4-trimethyl-3-(3,8,12,16-tetramethyl-heptadeca-3,7,11,15-tetraenyl)-cyclohexanol) triterpenoid. Diterpenoids isolated from *A. laxiflora* leaves’ methanol and petroleum ether extracts are 17-hydroxyingenol and 3,7,11,15-tetramethyl-2-hexadecen-1-ol (Sandjo et al., 2011; Tapondjou et al., 2016; Okokon et al., 2017b; 2017a; Morah and Uduagwu, 2017; Bafor et al., 2018). Morah and Uduagwu (2017) investigated five carotenoid pigments (astaxanthin, lycoxanthin, rhodopin, dimethoxy-lycopene, and anhydrorhodovibrin), one sesquiterpene (hexahydrofarnesyl acetone), and five steroids (glycocholic acid, ethyl iso-allocholate, 7,8-epoxylanostan-11-ol, 3-acetoxy-, and 4-vinylcholestan-3-ol) from leaves of *A. laxiflora* using petroleum ether and ethanol fraction and structure confirmed by GS-MS. Sandjo et al. (2011) and Tapondjou et al. (2016) isolated and established the structure of a known steroidal glycoside, β-sitosterol-3-O-β-D-glucopyranoside from the methanol extract of stem bark and leaves, respectively. Likewise, another steroid cholest-4-en-3-one was isolated and characterized by Okokon et al. (2017a) from *A. laxiflora* ethanol root extract. In an attempt to isolate some antimalarial and antiplasmodial constituents from *A. laxiflora*, leaves and root ethanol extract on fractionation resulted in the isolation of three terpenoids, namely, 2,6,10-trimethylundecan-(5*E*)-2,5,9-trien-4-one; 3,7,11,15-tetramethyl-2-hexadecen-1-ol and 2(4*H*)-benzofuranone, 5,6,7,7A-tetrahydro-6-hydroxy-4,4,7- (Okokon et al., 2017b; 2017a).

### Fatty acids

Recently, essential oil from *A. laxiflora* leaves hydro-distillate on GC-MS analysis offered three long-chain aliphatic acids (palmitic, oleic, and petroselinic) (Otuechere et al., 2019). In another study, the crude methanolic extract of *A. laxiflora* leaves was subjected to column chromatographic separation and HR-ESI-TOF-MS analysis, resulting in the isolation of one novel fatty acid ester, namely, (10*Z*)-tetradec-10-enoic acid-(2*S*)-2-carboxy-2-hydroxyethyl ester, and one new ceramide, (2*R*)-2-hydroxy-*N*-[(2*S*,3*S*,4*R*,15*Z*)-1,3,4-trihydroxy-15-triaconten-2-yl]octacosamide (Sandjo et al., 2011). Morah and Uduagwu (2017) studied the GC-MS of petroleum ether and ethanol extracts of the *A. laxiflora* leaves and identified twelve fatty acid derivatives, namely, ethyl linoleate, icosyl oleate, oleyl palmitoleate, methyl palmitate, ethyl palmitate, cyclopropanedodecanoic acid, 2-octyl-, methyl ester; methyl isostearate, 1-heptatriacotanol; ethanol, 2-(9,12-octadecadienyloxy)-, (*Z*,*Z*)-; 9-octadecene, 1,1′-[1,2-ethanediylbis(oxy)] bis-, (*Z*,*Z*)-; 9-desoxy-9x-chloroingol 3,7,8,12-tetraacetate and one lactone fatty acid ester tricyclo [20.8.0.0(7,16)]triacontane, 1(22),7(16)-diepoxy- (Table 2; Supplementary Figure S4). Moreover, Okokon et al. (2017b) also isolated thirteen saturated and unsaturated fatty acids and esters, namely, pentadecanoic acid, stearic acid, 2-hydroxyethyl oleate, henicosyl formate, methyl ricinoleate, methyl linoleate, methyl elaidolinolenate, 1,3-diacetyloxypropan-2-yl icosanoate, 1-tetradecanol, 1-hexadecanol, dimethyl undecanedioate, 1-heptacosanol, and *Z*,*E*-2,13-octadecadien-1-ol. Okokon et al. (2017a) isolated and characterized a multitude of fatty acids from *A. laxiflora* root ethyl acetate fraction, including ethyl oleate, methyl oleate, α-linoleic acid, propyl linoleate, methyl palmitate, ethyl palmitate, trimethylsilyl palmitate, stearic acid, ethyl stearate, ethyl tetracosanoate, pentadecanoic acid, ethyl ester, ethyl laurate, ethyl myristate, elaidic acid trimethylsilyl, and a lactone fatty acid ester 2*H*-pyran-2-one, tetrahydro-4-hydroxy-6-pentyl (Supplementary Figure S5 **)**.

### Alkaloids

Various studies reported the presence of alkaloids in *A. laxiflora* using phytochemical screening, but only a few compounds have been isolated (Table 2; Supplementary Figure S6). An unusual prenylguanidinyl-epicatechin derivative alchornealaxine was separated from *A. laxiflora* leaves by Tapondjou et al. (2016). In another report, one pyrrolidine alkaloid, namely, 4-fluoro-2-nitroaniline, 5-[4-(pyrrolidin-1-yl) carbonylmethylpiperazin1-yl]-, was isolated from the extract of *A. laxiflora* leaves (Morah and Uduagwu, 2017). In two distinct studies by Okokon et al. (2017b, 2017a), two capsaicinoid alkaloids (capsaicin and dihydrocapsaicin) and one purine alkaloid called 1,3,7,9-tetramethyluric acid were isolated from the leaves and roots of *A. laxiflora.* Bafor et al. (2018) captured the porphine derivative pheophorbide A from the methanol *A. laxiflora* extract.

### Miscellaneous compounds

Notable compounds isolated from *A. laxiflora* include megastigmane glycoside (byzantionoside B, leeaoside), carbohydrates (2-methylerythritol, 4-amino-4-deoxyarabinose, 3-deoxy-arabino-hept-2-ulosonic acid), amino acids (2-amino-4,5-dihydroxy-3,4-dimethylpentanoic acid), alkanes (octadecane, 3-ethyl-5-(2-ethylbutyl)-), and 2-methyl-3,5-dinitrobenzyl alcohol (Tapondjou et al., 2016; Morah and Uduagwu, 2017). Two distinct studies by Okokon et al. (2017b, 2017a) reported the GC and GC-MS analysis of ethyl acetate fraction from *A. laxiflora* leaves and roots. They discovered that the ethyl acetate fraction was dominated by multifarious bioactive compounds: 1-hexadecene, 1-octadecene, 1-tetradecene, D-galactitol,3,6-anhydro-1,2,4,5-tetra-O-methyl-; 10,11-dihydro-10-hydroxy-2,3-dimethoxydibenz(b,f)oxepin; hydroxy-4,4-dimethyldihydro-2(3*H*)-furanone; 2-coumaranone, 2-cyclopenten-1-one, 2-methyl-; (*Z*),(*Z*)-2,5-dimethyl-2,4-hexadienedioic acid; 1-octadecene, propanoic acid, 3-(trimethylsilyl)-, ethyl ester; 2-furancarboxylic acid, trimethylsilyl ester; cyclopropenoic acid,1-trimethylsilyl,-2-(2-methylpropen-1-yl), methyl ester; 2*H*-pyran-2-one, 5,6-dihydro-6-pentyl-, (R)-; 1,2,4-cyclopentanetrione, 3-butyl-; octadecane, 1-bromo-; 2-butenoic acid, 2-methoxy-3-methyl-, methyl ester; benzoic acid, 3-acetyloxy-, trimethylsilyl ester; cyclopropanecarboxylic acid, 2,2-dimethyl-3-cis-(2-methyl-3-buten-2-yl)-; 3-{[tert-butyl (dimethyl)silyl]oxy}butanal; benzeneacetic acid, alpha-[(trimethylsilyl)oxy]-; 2-hydroxy-3-methoxybenzaldehyde, trimethylsilyl ether and benzene, and (2-ethyl-4-methyl-1,3-pentadienyl)-, (E) (Table 2; Supplementary Figure S7).

This literature review reveals that *A. laxiflora* is rich in flavonoids, phenolic compounds, terpenoids, fatty acids, steroids, and alkaloids. To date, 132 compounds have been identified and structurally elucidated from the extracts of *A. laxiflora,* including 13 flavonoids, 19 phenolics, 22 terpenoids, 43 fatty acids, 6 alkaloids, and other secondary metabolites. Nonetheless, most of these compounds have been identified from the leaves, stems, and roots of this plant. Consequently, it is suggested to utilize inflorescence, flowers, and fruits to identify and isolate chemical constituents. Moreover, further research is required to establish the therapeutic applications of isolated compounds of this plant.

## Pharmacological activity

**FIGURE 4:**
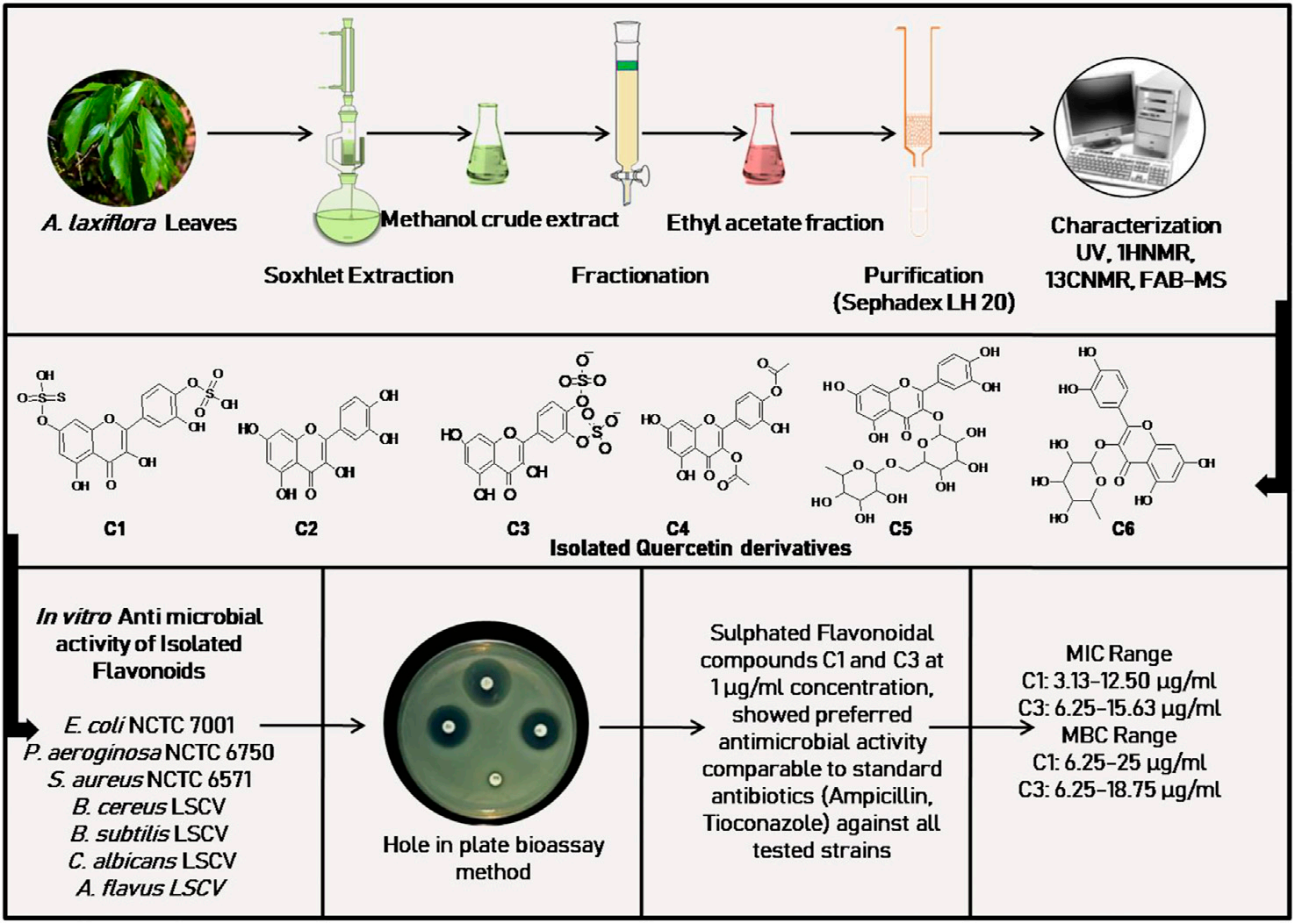
Anti-microbial activity of isolated flavonoids from *A. laxiflora*, derived from Ogundipe et al. (2001b).

**FIGURE 5:**
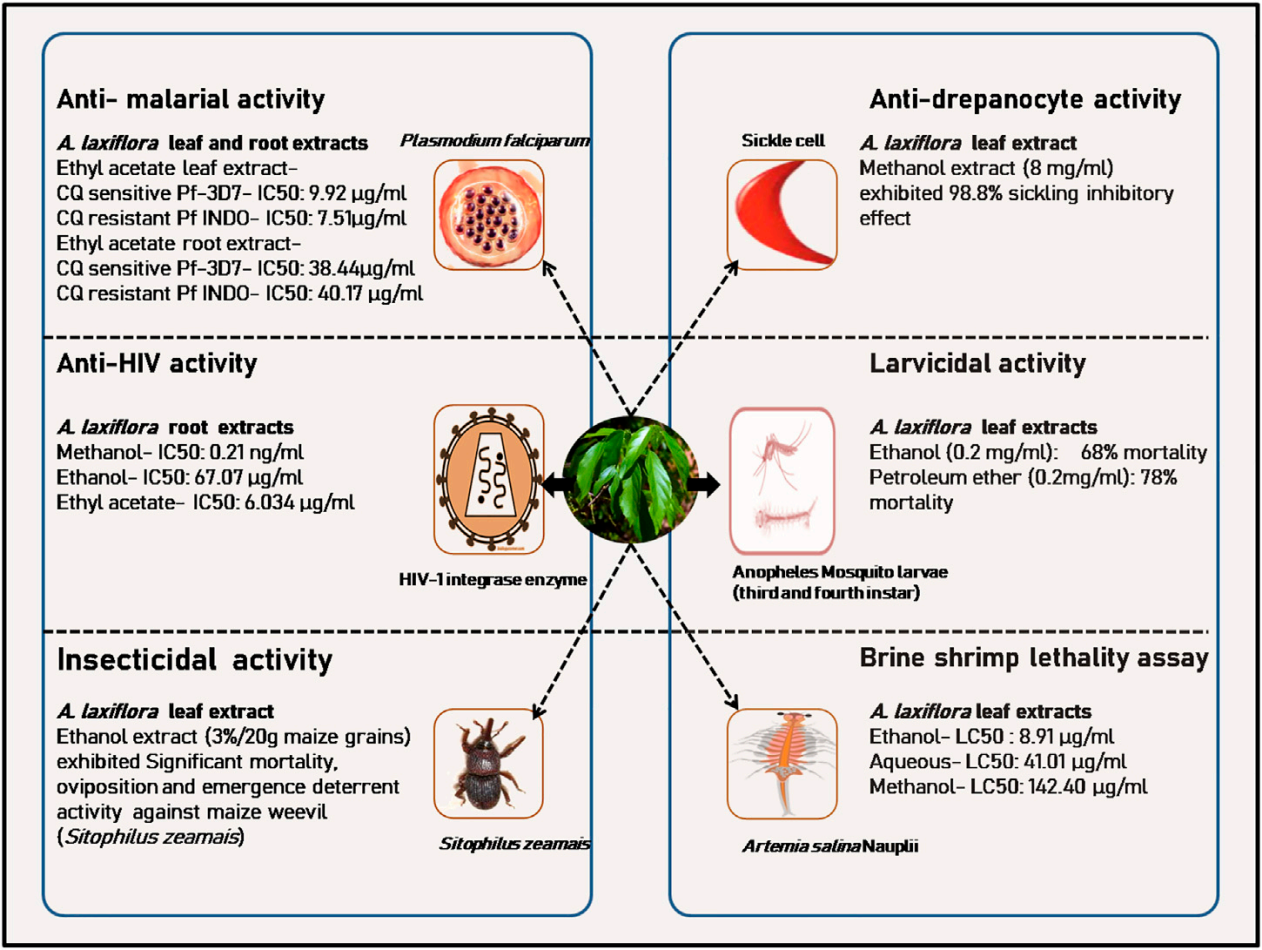
A few *in vitro* pharmacological and toxicological activities of *A. laxiflora* derived from Ogbole et al. (2016), Okokon et al. (2017a; 2017b), Morah and Uduagwu (2017), Osabiya et al. (2017), Bamimore and Elujoba (2018), Siwe-Noundou et al. (2019), and Ileke et al. (2020).

**FIGURE 6:**
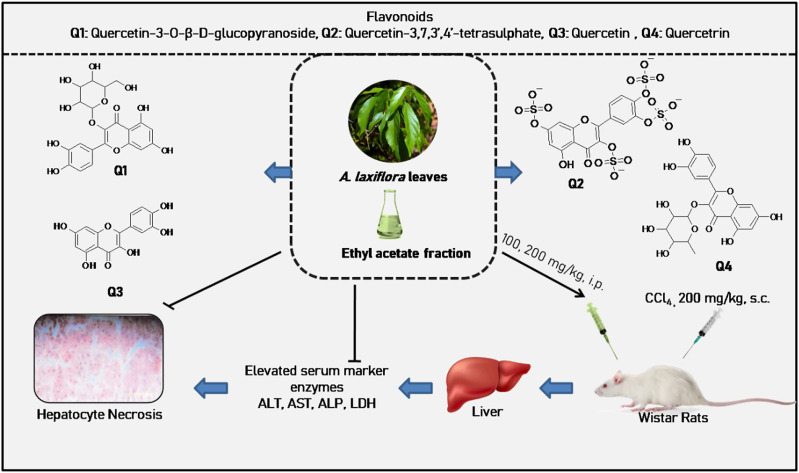
Hepatoprotective activity of *A. laxiflora* derived from Oloyede et al. (2011).

Traditionally, the whole plant, leaves, roots, stem, and fruits of *A. laxiflora* are used to treat various complications in different regions of Africa (Figure 2). In particular, the Cameroonian traditional medicine system documented different applications of this plant as remedies for various health issues in Cameroonian traditional pharmacopeia (Sandjo et al., 2011). A wide range of pharmacological activities of the *A. laxiflora* extracts and its isolated phytochemicals have been reported using different *in vitro* and *in vivo* methods in the last 2 decades (Tables 3, 4 and Figures 4–6).

**TABLE 3:** *In vitro* anti-microbial activity of the extract, fractions, and compounds of *A. laxiflora*.

Extracts/compounds	Model	MIC/ZOI	Concentration	References
C1, C2, C3, C4, C5, and C6	*Pseudomonas aeruginosa* (*P. aeruginosa*) NCTC 6750	6.25, 97.13, 12.50, NS, 125.2. ns μg/ml, respectively	1 mg/ml	Ogundipe et al. (2001b)
	*Staphylococcus aureus* (*S. aureus*) NCTC 6571	3.13, 62.50, 6.25, 62.5, 75, ns μg/ml, respectively		
	*Bacillus cereus* (*B. cereus*) LSCV	3.13, 75, 12.5, 62.5, 75, NS μg/ml, respectively		
	*Candida albicans* (*C. albicans*) LSCV	12.50, 125, 15.63, 97.13, 109.6, NS μg/ml, respectively		
	*Aspergillus flavus* (*A. flavus*) LSCV	ND		
	*Escherichia coli* (*E. coli*) NCTC 7001	ND		
	*Bacillus subtilis* LSCV	ND		
ELE	*S. aureus* NCIB 8588, *B. subtilis* NCIB 3610, *E. coli* NCIB 86, *Proteus vulgaris* (*P. vulgaris*) NCIB 67, *P. aeruginosa* NCIB 950, *Klebsiella pneumoniae* (*K. pneumoniae*) NCIB 418, *C. albicans* (clinical), and *A. flavus* (clinical)	250, >250, and 250 μg/ml against *P. aeruginosa*, *P. vulgaris*, and *A. flavus*, respectively	6.25–250 μg/ml	Essiett and Ajibesin (2010)
MLE	*S. aureus* NCIB 8588, *S. aureus* SW1, *S. aureus* SW2, *S. aureus* SS1, *S. aureus* SS2, *S. aureus* SS3, *S. aureus* NC1, *S. aureus* NC2, *S. aureus* NC3, *S. aureus* NC4, *Micrococcus luteus* NCIB 196, *Pseudomonas fluorescens* NCIB 3756, *B. cereus* NCIB 6349, *Clostridium sporogenes* NCIB 532, *Shigella* species ST1, *Shigella* species ST2, *Shigella* species ST3, *Bacillus stearothermophilus* NCIB 8222, *E. coli* NCIB 86, *E. coli* ST1, *E. coli* ST2, *E. coli* ST3, *K. pneumoniae* NCIB 418, *K. pneumoniae* SS1, *K. pneumoniae* SS2, *K. pneumoniae* SS3, *B. subtilis* NCIB 3610, *Bacillus polymyxa* ES, *Clostridium pyogenes* ES, *Enterococcus faecalis* (*E. faecalis*) NCIB 775, *P. vulgaris* ES1, *P. aeruginosa* ES2, *P. aeruginosa* ES3, *P. aeruginosa* ES4, *P. aeruginosa* ES5, *P. aeruginosa* ES6, *P. aeruginosa* ES7, *P. aeruginosa* ES8, and *Bacillus anthracis* ES	1.56, 1.56, 3.13, 3.13, 1.56, 1.56, 12.50, 3.13, 3.13, 6.25, 1.56, 1.56, 3.13, 3.13, 3.13, 3.13, 3.13, 3.13, 25, 25, 25, 25, 3.13, 3.13, 25, 25, 1.56, 0.78, 3.13, 6.25, 1.56, 3.13, 12.50, 12.50, 12.50, 12.50, 25, 12.50, and 6.25 mg/ml, respectively against tested strains	0.78–25 mg/ml	Akinpelu et al. (2015)
MLE	*Aspergillus niger*, *Aspergillus fumigatus*, *Aspergillus glaucus*, *Fusarium species*, *Penicillium expansum*, *Alternaria species*, *Trichophyton tonsurans* (*T. tonsurans*), *Trichophyton interdigitale*, *Penicillium camemberti*, *Trichophyton mentagrophytes*, *Trichoderma species* (*nonpathogen*), *A. flavus*, *Scopulariopsis brevicaulis*, *Penicillium italicum*, *Trichophyton rubrum*, *C. albicans*, and *Candida pseudotropicalis*	35, 35, 17.50, ND, 17.50, 35, 2.19, 17.50, 17.50, 17.50, 8.75, 35, 35, 8.75, ND, 35, 8.75 mg/ml, respectively, against tested strains	2.19–35 mg/ml	Akinpelu et al. (2015)
TMA	*E. coli* (ATCC 8739, AG102, AG100 A_tet_), *Enterobacter aerogenes* (*E. aerogenes*) (ATCC 13048, CM64, EA27), *K. pneumoniae* (ATCC 11296, KP55), *Providencia stuartii* (*P. stuartii*) (ATCC29916, PS299645), *E. cloacae* (BM47, BM67), and *P. aeruginosa* (PA01, PA124)	256, >256, >256, >256. >256, >256, 256, >256, 64, 256, >256, >256, ND, ND, µg/ml, respectively, against tested pathogens	NS	Mbaveng et al. (2015)
MLE	*E. coli* (ATCC8739, ATCC10536, AG100ATet, AG102), *E. aerogenes* (ATCC13048, CM64, EA 27, EA 289), *K. pneumoniae* (ATCC11296, KP55, KP63), *P. stuartii* (ATCC29916, NEA 16), and *P. aeruginosa* (PA01, PA124)	256, 128, >1024, 256, 512, 512, 128, 128, 256, 512, 512, >1,024, 128, 512, and >1,024 μg/ml, respectively, against tested strains	NS	Tchinda et al. (2017)
MSE	*E. coli* (ATCC8739, ATCC10536, AG100ATet, AG102), *E. aerogenes* (ATCC13048, CM64, EA 27, EA 289), *K. pneumoniae* (ATCC11296, KP55, KP63), *P. stuartii* (ATCC29916, NEA 16), and *P. aeruginosa* (PA01, PA124)	1,024, 512, >1,024, 512, 512, 512, >1,024, 64, 256, 512, >1,024, 512, >1,024, 512, and >1,024 μg/ml, respectively, against tested strains	NS	Tchinda et al. (2017)
MLE	*E. coli* ATCC 10536, *E. faecalis* ATCC 1054, *E. aerogenes* ATCC13048, *Shigella flexneri* (*S. flexneri*), *Salmonella typhi* (*S. typhi*) ATCC 6539, and *S. aureus*	1,024 and 512 μg/ml against *S. typhi* and *S. flexneri*, respectively; >1,024 μg/ml against other strains	8–1,024 μg/ml	Wansi et al. (2017)
ALE	*E. coli* ATCC 10536, *E. faecalis* ATCC 1054, *E. aerogenes* ATCC13048, *S. flexneri*, *S. typhi* ATCC 6539, and *S. aureus*	>1,024 μg/ml against all tested strains	8–1,024 μg/ml	Wansi et al. (2017)
ALE	*B. subtilis*, *S. aureus*, *E. coli*, *E. faecalis*, *K. pneumoniae*, *S. typhi*	25, 10, 10, 2.5, 12.5, and 40 mg/ml, respectively, against tested strains	10, 20, 30, 40, and 50 mg/ml	Osabiya et al. (2017)
ELE	*B. subtilis*, *S. aureus*, *E. coli*, *E. faecalis*, *K. pneumoniae*, *S. typhi*	10, 5, 5, 5, 2.5, and 30 mg/ml, respectively, against tested strains	10, 20, 30, 40, and 50 mg/ml	Osabiya et al. (2017)
MMLE	*Helicobacter pylori (H. pylori)*	20 mg/ml	0.125, 0.25, 0.5, 1, 2, 10, 20, 50, and 100 mg/ml	Ngnameko et al. (2019)
HxRE, ChRE, EaRE, MRE, ERE, and ARE	*B. cereus* ATCC 11778	500, 63, 250, 63, 63, and 8,000 μg/ml, respectively	32 mg/ml	Siwe-Noundou et al. (2019)
	*E. faecalis* ATCC 29212	63, 50, 2,000, 63, 63, and 8,000 μg/ml, respectively		
	*E. coli* ATCC 25922	1,000, 500, 500, 500, 500, and 8,000 μg/ml, respectively		
	*S. aureus* ATCC 25923	63, 50, 50, 50, 50, and 8,000 μg/ml, respectively		
	*K. pneumoniae* ATCC 13883	2,000, 125, 125, 125, 125, and 8,000 μg/ml, respectively		
	*Moraxella catarrhalis* (*M. catarrhalis*) ATCC 23246	2,000, 500, 1,000, 1,000, 500, and >8,000 μg/ml, respectively		
	*Proteus mirabilis* (*P. mirabilis*) ATCC 43071	1,000, 500, 250, 250, 250, and >8,000 μg/ml, respectively		
	*Staphylococcus saprophyticus* (*S. saprophyticus*) ATCC 15305	125, 63, 63, 63, 63, and >8,000 μg/ml, respectively		
HxSE, ChSE, EaSE, MSE, ESE, and ASE	*B. cereus* ATCC 11778	8,000, 250, 250, 1,000, 500, and 8,000 μg/ml, respectively	32 mg/ml	Siwe-Noundou et al. (2019)
	*E. faecalis* ATCC 29212	8,000, 2,000, 500, 1,000, 1000, and 8,000 μg/ml, respectively		
	*E. coli* ATCC 25922	8,000, 500, 250, 500, 250, and 8,000 μg/ml, respectively		
	*S. aureus* ATCC 25923	1,000, 500, 500, 500, 500, and 8,000 μg/ml, respectively		
	*K. pneumoniae* ATCC 13883	2,000, 500, 500, 500, 500, and 4,000 μg/ml, respectively		
	*M. catarrhalis* ATCC 23246	>8,000, 2,000, 2,000, 500, 500, and >8,000 μg/ml, respectively		
	*P. mirabilis* ATCC 43071	>8,000, 2,000, 8,000, 4,000, 4,000, and >8,000 μg/ml, respectively		
	*S. saprophyticus* ATCC 15305	250, 250, 250, 63, 63, and >8,000 μg/ml, respectively		
HLE, ChLE, EaLE, MLE, ELE, and ALE	*B. cereus* ATCC 11778	500, 125, 125, 125, 125, and 4,000 μg/ml, respectively	32 mg/ml	Siwe-Noundou et al. (2019)
	*E. faecalis* ATCC 29212	500, 125, 125, 250, 250, and 1,000 μg/ml, respectively		
	*E. coli* ATCC 25922	500, 125, 125, 125, 125, and 4,000 μg/ml, respectively		
	*S. aureus* ATCC 25923	250, 250, 250, 250, 400, and 1,000 μg/ml, respectively		
	*K. pneumoniae* ATCC 13883	1,000, 500, 63, 63, 63, and 8,000 μg/ml, respectively		
	*M. catarrhalis* ATCC 23246	1,000, 1,000, 125, 2,000, 1,000, and >8,000 μg/ml, respectively		
	*P. mirabilis* ATCC 43071	>8,000, 8,000, 8,000, 8,000, 2,000, and >8000 μg/ml, respectively		
	*S. saprophyticus* ATCC 15305	250, 63, 63, 63, 250, and >8,000 μg/ml, respectively		
EA, MA, GS, AOA, and AUA	*B. cereus* ATCC 11778	125, 125, 125, 125, and 125 μg/ml, respectively	1 mg/ml	Siwe-Noundou et al. (2019)
	*E. faecalis* ATCC 29212	63, 63, 63, 125, and 125 μg/ml, respectively		
	*E. coli* ATCC 25922	63, 63, 63, 63, and 63 μg/ml, respectively		
	*S. aureus* ATCC 25923	125, 125, 125, 125, and 125 μg/ml, respectively		
	*K. pneumoniae* ATCC 13883	16, 31, 31, 16, and 31 μg/ml, respectively		
	*M. catarrhalis* ATCC 23246	125, 250, 125, 16, and 16 μg/ml, respectively		
	*P. mirabilis* ATCC 43071	125, 250, 250, 63, and 63 μg/ml, respectively		
	*S. saprophyticus* ATCC 15305	31, 16, 4, 4, and 4 μg/ml, respectively		
LEO	*B. subtilis* ATCC 6633, *B. cereus* ATCC 10872, *E. coli* ATCC 25922, *P. aeruginosa* ATCC 9027, and *S. aureus* ATCC 25922	ZOI: 8, 9, 9, 4, and 7 mm, respectively	100 mg/ml	Otuechere et al. (2019)
ALE, EaLE, and ELE	*S. typhi*	ZOI ranged 6–8, 12–24, and 4–8 mm for aqueous, ethyl acetate, and ethanol, respectively	20, 40, and 60 mg/ml	Osuntokun and Olajubu (2015)
	*Salmonella paratyphi*	ZOI ranged 7–11, 9–19, and 6–10 mm for aqueous, ethyl acetate, and ethanol, respectively		

C1: quercetin-7,4′-disulphate, C2: quercetin, C3: quercetin-3′,4′-disulphate, C4: quercetin-3,4′-diacetate, C5: rutin, C6: quercitrin, SW: surgical wound isolate, SS: sepsis wound isolate, NC: nasal cavity isolate, ST: stool isolate, ES: environmental isolate, TMA: 3,4,3′-tri-O-methylellagic acid, ALE: aqueous leaf extract, EaLE: ethyl acetate leaf extract, ELE: ethanol leaf extract, LEO: essential oil from leaf, EA: ellagic acid, MA: 3-O-methylellagic acid, GS: 3-O-β-D-glucopyranosyl-β-sitosterol, AOA: 3-O-acetyl-oleanolic acid, AUA: 3-O-acetyl-ursolic acid, MLE: methanol leaf extract, HLE: hexane leaf extract, ChLE: chloroform leaf extract, HxSE: hexane stem bark extract, ChSE: chloroform stem bark extract, ASE: aqueous stem bark extract, EaSE: ethyl acetate stem bark extract, MSE: methanol stem bark extract, ESE: ethanol stem bark extract, MMLE: methylene chloride/methanol (1:1; v/v) leaf extract, HxRE: hexane root extract, ChRE: chloroform root extract, EaRE: ethyl acetate root extract, MRE: methanol root extract, ERE: ethanol root extract, ARE: aqueous root extract, NS: not specified, ND: not determined.

**TABLE 4:** Pharmacological activities of extracts/fractions and compounds of *A. laxiflora*.

Activity	Extract/compounds	Model	Effects/activity	Study	Dosage	References
Anti-amoebic activity	MMLE	*E. histolytica* clinical isolate	Mild anti-amoebic activity	*In vitro*	100 μg/ml	Moundipa et al. (2005)
Anti-malarial activity	ELE, PELE, CHLE, EALE, BLE, and ALE	CQ sensitive Pf-3D7	IC_50_: 31.57 ± 0.94, 27.85 ± 0.36, 26.06 ± 0.19, 9.92 ± 0.28, >100, >100 μg/ml, respectively	*In vitro*	NS	Okokon et al. (2017b)
		CQ-resistant Pf INDO	IC_50_: 16.38 ± 0.94, 23.47 ± 0.15, 14.47 ± 0.35, 7.51 ± 0.24, 52.63 ± 0.22, and >100 μg/ml, respectively			
	ELE	*P. berghei*-infected Swiss albino mice	Showed dose-dependent but weak suppressive, repository, and schizonticidal activity compared to standard antimalarial drugs	*In vivo*	200, 400, and 600 mg/kg	
	ERE, PERE, DMRE, EaRE, BRE, and ARE	CQ sensitive Pf-3D7	IC_50_: 52.73 ± 2.26, 81.20 ± 2.34, 72.72 ± 1.14, 38.44 ± 0.89, >100, and >100, respectively, against tested extract and fractions	*In vitro*	NS	Okokon et al. (2017a)
		CQ-resistant Pf INDO	IC_50_: 56.71 ± 3.43, 90.24 ± 3.38, 73.48 ± 2.35, 40.14 ± 0.78, 98.99 ± 1.53, and >100, respectively, against tested extracts and fractions			
		*P. berghei*-infected Swiss albino mice	Showed dose-dependent but weak suppressive, repository, and schizonticidal activity compared to standard antimalarial drugs	*In vivo*	200, 400, and 600 mg/kg	
	MLE, CHLE	*P. berghei*-infected Swiss albino mice	Significant dose-dependent suppressive, repository, and schizonticidal activity	*In vivo*	200, 400, and 600 mg/kg	Oluyemi and Blessing (2019)
Anti-inflammatory activity	ACLE	Soybean 15- LOX inhibition assay	IC_50_: 46.03 ± 2.10	*In vitro*	100 μg/ml	Dzoyem and Eloff (2015)
		LPS activated RAW 264.7 cell (NO production inhibition)	86.38%, 90.96%, and 96.53% inhibition, respectively, at tested dose		6.25, 12.5, and 25 μg/ml	
	MMLE	Soybean 15- Lox inhibition assay	54.58 ± 2.39% inhibition; IC_50_ 90.42 ± 0.42	*In vitro*	100 μg/ml	Ndam Ngoungoure et al. (2019)
	MMLE	LPS activated RAW 264.7 cell (NO production inhibition)	68.10 ± 1.64%; IC_50_ 66.57 ± 4.01			
Analgesic activity	ERE, DMF, EAF, and BF	Acetic acid-induced writhing, formalin-induced paw licking, and thermally induced pain in mice	ERE and EAF showed significant analgesic activity in all models compared to standard drug	*In vivo*	75, 150, and 225 mg/kg	Okokon et al. (2017a)
	ALE, MLE	Hot plate and tail immersion tests in mice	Showed significant analgesic activity in both animal models. Higher doses (800 and 1,600) showed better analgesic activity than lower doses	*In vivo*	100, 200, 400, 800, and 1,600 mg/kg	Nwonu et al. (2018a)
Anti-diabetic activity	MLE	Alpha-amylase inhibitory assay	IC_50_: 295.60 ± 0.53 μg/ml	*In vitro*	31.25–1,000 μg/ml	Ogbole et al. (2016)
	MLE	Alloxan-induced diabetic rat model	Significantly lowered blood glucose level in diabetic rats	*In vivo*	500 mg/kg	Nimenibo-uadia, (2018)
Anti-HIV activity	HRE, CHRE, EaRE, MRE, ERE, ARE, and MSE	HIV-1 integrase strand transfer assay	IC_50_: ND, ND, 6.034, 0.0002083, 0.06707, >500 and ND, respectively, against tested extracts	*In vitro*	25 μg/ml	Siwe-Noundou et al. (2019)
	EA, MA, GS, AOA, and AUA	HIV-1 integrase strand transfer assay	IC_50_: 90.23, >100, ND, >100 and ND, respectively, against tested compounds		20 µM	
Larvicidal activity	ELE	*Anopheles* larva. Larvicidal bioassay	Mortality: 32%, 38%, 60%, and 68%, respectively, at assayed concentrations	*In vitro*	0.08, 0.1, 0.15, and 0.2 mg/ml	Morah and Uduagwu, (2017)
	PELE	*Anopheles* larva. Larvicidal bioassay	Mortality: 30%, 38%, 60%, and 68%, respectively, at assayed concentrations			
Anti-Parkinson’s disease activity	MMLE	Aminochrome-induced toxicity in human astrocytoma cells (U373MG and U373MGsiGT6)	Significantly decreased aminochrome-induced toxicity in both cell lines	*In vitro*	0.1–1 μg/ml	Ngoungoure et al. (2019)
Anti-psychotic activity	ALE and MLE	Apomorphine-induced climbing behavior and stereotypic behavior; mice	Dose-dependent significant reduction in climbing and stereotypy behaviors	*In vivo*	100, 200, 400, 800, and 1,600 mg/kg	Nwonu et al. (2018c)
Anti-Alzheimer activity	ACLE	AChE inhibitory assay	IC_50_: 364.12 ± 2.39 μg/ml	*In vitro*	0.007, 0.016, 0.031, 0.063, and 0.125 mg/ml	Dzoyem and Eloff (2015)
	MMLE	AChE inhibition assay	36.02 ± 0.18% AchE inhibition, IC_50_: >200 μg/ml	*In vitro*	200 μg/ml	Ngoungoure et al. (2019)
	HxSE, EaSE, and AqSE	AChE and BuChE inhibition assay	%Inhibition	*In vitro*	NS	Elufioye (2017)
			AChE: 12.31%, 28.10%, 10.69%			
			BuChE: 4.02%, 16.60%, 13.33%			
	HRE, EaRE, and ARE		%Inhibition			
			AChE: 13.10%, 25.04%, 12.55%			
			BuChE: 18.46%, 15.68%, 13.88%			
	HLE, EALE, and ALE		%Inhibition			
			AChE: 10.69%, 34.20%, 17.38%			
			BuChE: 7.73%, 18.15%, 4.88%			
Anti-convulsant activity	ALE	Swiss albino mice PIC, PTZ, INH, STR, NMDA, MES-induced convulsion test	At 60 mg/kg dose protected against NMDA-induced turning behavior and at 120 mg/kg protected 75% mice in STR-induced convulsions, no effect against PTZ, MES, PIC, and INH-induced convulsions	*In vivo*	12, 30, 60, and 120 mg/kg	Bum et al. (2009)
Sedative Activity	ALE	Diazepam-induced sleep in mice	Failed to produce sedative action at all tested dose	*In vivo*	12, 30, 60, and 120 mg/kg	Bum et al. (2009)
Anxiolytic activity	ALE and MLE	Elevated plus maze and staircase exploratory behavior in mice	Significantly increased the percent entry into open arms and increased the percent time spent in open arms in the elevated plus maze test and a significant decrease in rearing and increase in the number of steps climbing in staircase exploratory test	*In vivo*	100, 200, 400, 800, and 1,600 mg/kg	Nwonu et al. (2018b)
Anti-diarrheal activity	ALE and MLE	*S. flexneri*, castor, magnesium-induced diarrhea in rats	Methanolic extract showed a significant antidiarrheal effect in all models	*In vivo*	125, 250, and 500 mg/kg	Wansi et al. (2017)
Anti-anemia activity	ALE	Iron deficient rats	Significantly increased hematological indices (Hb, RBC, MCV, MCH, and MCHC) at all tested dose	*In vivo*	100, 200, and 300 mg/kg	Oladiji et al. (2014)
	ELE	Male albino rats	The extract significantly increased all hematological indices (RBC, WBC, PCV, platelet, and Hb) at all the dose assayed	*In vivo*	100, 200, and 300 mg/kg	Bada et al. (2017)
	ALE	Iron deficient rats	Significantly reversed the anemic condition in iron-deficient rats by increasing disaccharidases activity and gastric pH at all tested dose	*In vivo*	100, 200, and 300 mg/kg	Soladoye et al. (2014)
	MLE	Inhibitory and reversal anti-sickling assay	Extract at 8 mg/ml showed the highest 98.8% sickling inhibitory effect and at 4 mg/ml marginally reversed the sickling of Hb (48.66%)	*In vitro*	2, 4, 6, and 8 mg/ml	Bamimore and Elujoba (2018)
Antioxidant activity	HRE, MRE, MLE, and HLE	Thiocyanate assay	Antioxidant activity order: HRE (76.4%) > MRE (63%) > MLE (40%) > HLE (38%)	*In vitro*	NS	Farombi et al. (2003)
		ABTS assay	Total antioxidant activity: 8, 6.5, 5, and 3 mM equivalent of ascorbic acid, respectively		2.5 mg/ml	
	HRF: FI, FII, FIII, FIV, FV, and FVI	Lipid peroxidation (TBARS)	48%, 69%, 16%, 11%, 5%, and 44% inhibition		1 mg/ml	
	ELE, EaF, and BuF	DPPH assay	EC_50_: 12.97, 24.34, and 106.74 μg/ml for EaF, BuF, and ELE, respectively	*In vitro*	2.5, 5, 10, 25, 50, 125, and 250 μg/ml	Adeloye et al. (2005)
	HLE, EALE, BLE, and ALE	Ferric thiocyanate method	All extracts at 500 μg/ml showed antioxidant activity (70%–78%) compared to vitamin E (82%)	*In vitro*	50, 100, 250, and 500	Oloyede et al. (2010)
					µg/ml	
	ACLE	DPPH assay	IC_50_: 17.19 ± 1.02 μg/ml	*In vitro*	NS	Dzoyem and Eloff, (2015)
		ABTS assay	IC_50_: 18.53 ± 1.42 μg/ml			
		FRAP assay	IC_50_: 438.42 ± 15.55 μg/ml			
	MLE	Wistar rats	Extract exhibited potent elevation of antioxidant enzymes: serum CAT and SOD level in a dose-dependent manner and liver GSH level at 0.5 and 50 mg/kg	*In vivo*	0.5, 1, 10, and 50 mg/kg	Uhunmwangho et al. (2017)
	PELE	DPPH assay	Radical scavenging ability: 39.24%, 41.12%, 42.01%, 46.84%, and 50.50% at 0.04, 0.08, 0.1.0.15, and 0.2 mg/ml, respectively	*In vitro*	0.04, 0.08, 0.1, 0.15, and 0.2 mg/ml	Morah and Uduagwu (2017)
	ELE	DPPH assay	Radical scavenging ability: 8.32%, 12.68%, 24.13%, 37.76%, and 42.95% at 0.04, 0.08, 0.1.0.15, and 0.2 mg/ml, respectively			
Hepatoprotective activity	MMLE	Male Wistar rats, liver microsomal lipid peroxidation, and protein oxidation inhibition assay	Inhibition percent	*Ex vivo*	10, 100, and 200 μg/ml	Njayou et al. (2008)
			Non-enzymatic lipid peroxidation: 58.07 ± 9.91, 84.39 ± 0.75, and 95.90 ± 0.57			
			Enzymatic lipid peroxidation: 40.84 ± 0.39, 65.42 ± 1.77, and 79.17 ± 1.57			
			Protein oxidation: 58.40 ± 0.40, 85.61 ± 0.40, and 95.60 ± 0.59, respectively, at 10, 100, and 200 μg/ml concentrations			
	EALE	CCl_4_-induced hepatotoxicity in Wistar rats	The extract at 100 mg/kg significantly lowered the elevated serum levels of ALT, AST, AP, and LDH; reduction in centrilobular necrosis, vacuolization, and macrovesicular fatty changes in the liver at both doses	*In vivo*	100 and 200 mg/kg	Oloyede et al. (2011)
	HLE	Sodium arsenate-induced liver toxicity in albino rats	Pretreatment of extract exhibited better liver protection compared to the post-treatment group; the extract significantly decreased serum and liver biomarkers levels (AST, ALT, ALP, GGT, and TB) in a dose-dependent manner	*In vivo*	0.5, 1.0, 5, and 10 mg/kg	Esosa et al. (2013)
	MLE	CCl_4_-induced hepatotoxicity in Wistar rats	The extract caused a significant decrease in the liver marker enzymes (GGT, GST, ALT, and ALP) in a dose-dependent manner, with the highest activity at 50 mg/kg	*In vivo*	0.1, 0.5, 1.0, 10.0, and 50 mg/kg	Uhunmwangho et al. (2016)
	HRE	Sodium arsenate-induced liver toxicity in male Wistar rats	Pretreatment with extract reduced the elevated levels of liver markers (AST, ALT, and ALP), induced liver metabolizing enzymes (4-nitroanisole demethylase, glutathione-S-transferase, and cytochrome b_5_), total protein, albumin and globulin levels	*In vivo*	0.1, 0.5, 1.0, 10, 50, and 100 mg/kg	Uhunmwangho et al. (2018)
Anti-cancer activity	TChe, HtTO, AOA, AUA, MA, and MARp	HL-60 cells, MTT assay	IC_50_: 58.7, >100, 6.6, 6.8, >100, and >100 µM	*In vitro*	NS	Sandjo et al. (2011)
	MLE	Brine shrimp lethality assay	IC_50_: 142.40 μg/ml	*In vitro*	1.6–5,000 μg/ml	Ogbole et al. (2016)
	ELE, PeF	HeLa cells, MTT assay	TC_50_: 42.04, >100, 54.73, 8.83, >100, and >100 μg/ml	*In vitro*	100 μg/ml	Okokon et al. (2017b)
	ChF, EaF, BuF, and AqF	HEKS cells, MTT assay	TC_50_: 15.10, 23.32, 3.20, 1.41, 21.76, and >100 μg/ml, respectively, for tested extracts and fractions			
	MRE, MSE, and MLE	CCRF-CEM cells, resazurin reduction assay	IC_50_: >80, 49.21 ± 11.16, and 43.67 ± 4.06 μg/ml, respectively, for MRE, MSE, and MLE	*In vitro*	80 μg/ml	Kuete et al. (2016)
	ERE, PEF, DMF, EAF, BF, and AF	HeLa cells, MTT assay	Not cytotoxic; IC_50_: >100 μg/ml for all extracts and fractions	*In vitro*	100 μg/ml	Okokon et al. (2017a)
	ALE and ELE	Brine shrimp lethality assay	LC_50_: 8.91 and 41.01 for ELE and ALE, respectively	*In vitro*	1, 10, 100, 1,000 μg/ml	Osabiya et al. (2017)
	HRE, CHRE, EaRE, MRE, ERE, ARE, and MSE	HeLa cells, resazurin reduction assay	Not cytotoxic, percent viability was >100% against all extracts	*In vitro*	25 μg/ml	Siwe-Noundou et al. (2019)
	EA, MA, GS, AOA, and AUA	HeLa cells, resazurin reduction assay	Not cytotoxic, percent viability was >100% against all compounds		20 µM	
Tocolytic activity	MLE	Mice	The extract at 100 mg/kg exhibited progesterone-like effects on the ovaries, uterus, and cervical glands	*In vivo*	100 and 1,000 mg/kg	Bafor et al. (2015)
	MLE	Mice, spontaneous, oxytocin, and high KCl-induced uterine contraction inhibitory assay	Extract significantly inhibited uterine contractions in different assays	*Ex vivo*	0.0035 mg/ml, 0.035 mg/ml	Bafor et al. (2018)
					0.35 mg/ml and 3.5 mg/ml	
Fertility promoting effect	MLE	CCl_4_-induced reproductive toxicity in rats	The extract significantly reversed the toxic effects of CCl_4_ by increasing sperm motility and inhibiting sperm morphological aberrations	*In vivo*	0.1, 0.5, 1.0, 10.0, and 50 mg/kg	Uhunmwangho et al. (2016)

MMLE: methylene chloride/methanol (1:1; v/v) leaf extract, ELE: ethanol leaf extract, PELE: petroleum ether leaf extract, CHLE: chloroform leaf extract, EALE: ethyl acetate leaf extract, BLE: butanol leaf extract, ALE: aqueous leaf extract, ERE: ethanol root extract, PERE: petroleum ether root extract, DMRE: dichloromethane root extract, EaRE: ethyl acetate root extract, BRE: butanol root extract, ARE: aqueous root extract, MLE: methanol leaf extract, AcLE: acetone leaf extract, DMF: dichloromethane fraction of ethanol root extract, EAF: ethyl acetate fraction of ethanol root extract, BF: butanol fraction of ethanol root extract, HRE: hexane root extract, MSE: methanol stem bark extracts, ChRE: chloroform root extract, MRE: methanol root extract, EA: ellagic acid, MA: 3-O-methylellagic acid, GS: 3-O-β-D-glucopyranosyl-β-sitosterol, AOA: 3-O-acetyl-oleanolic acid, AUA: 3-O-acetyl-ursolic acid, HLE: hexane leaf extract, TChe: (10Z)-tetradec-10-enoic acid-(2S)-2-carboxy-2-hydroxyethyl ester; HtTO: (2R)-2-hydroxy-N-[(2S,3S,4R,15Z)-1,3,4-trihydroxy-15-triaconten-2-yl]octacosamide, MARp: 3-O-methylellagic acid-3′-O-α-rhamnopyranoside, HxSE: hexane stem bark extract, EaSE: ethylacetate stem bark extract, AqSE: aqueous stem bark extract, HRF: hexane root fractions, PeF: petroleum ether fraction of ethanol leaf extract, ChF: chloroform fraction of ethanol leaf extract, EaF: ethyl acetate fraction of ethanol leaf extract, BuF: butanol fraction of ethanol leaf extract, AqF: aqueous fraction of ethanol leaf extract, AF: aqueous fraction of ethanol root extract, PEF: petroleum ether fraction of ethanol root extract, ND: not determined, NS: not specified.

### Antibacterial and antifungal activity

The most common ethnomedicinal use of *A. laxiflora* includes the treatment of various infectious diseases such as typhoid, diarrhea, urinary tract infections, and venereal diseases. Hence, the application of *A. laxiflora* as an anti-infective agent prompted (Ogundipe et al., 2001b) to carry out bioactivity-guided isolation of the active constituent of *A. laxiflora*. Ogundipe et al. (2001b) evaluated six flavonoids associated with ethyl acetate fraction of methanolic *A. laxiflora* leave extract, namely, quercetin-7,4′-disulphate, quercetin, quercetin-3′,4′-disulphate, quercetin-3,4′-diacetate, rutin, and quercitrin against *Escherichia coli* (*E. coli*) NCTC 7001, *Pseudomonas aeruginosa* (*P. aeruginosa*) NCTC 6750, *Staphylococcus aureus* (*S. aureus*) NCTC 6571, *Bacillus cereus* (*B. cereus*) LSCV, *Bacillus subtilis* (*B. subtilis*) LSCV, *Candida albicans* (*C. albicans*) LSCV, and *Aspergillus flavus* (*A. flavus*) LSCV using the broth dilution method for determining the MIC and MBC of isolates with gentamicin 2.5 μg/ml, ampicillin 2.5 μg/ml, and tioconazole 10 μg/ml as reference compounds. The sulfated quercetin derivates quercetin-7,4′-disulphate (MIC range 3.13–12.50 μg/ml) and quercetin-3′,4′-disulphate (MIC range 6.25–15.63 μg/ml) showed preferred antimicrobial activity against all microorganism species than quercetin (MIC range 62.5–120.2 μg/ml) and comparable activity with standard drug ampicillin and tioconazole. Unfortunately, rutin and quercitrin did not exhibit any activity in this study (Table 3; Figure 4).

Different extracts (hexane, ethyl acetate, and butanol) of *A. laxiflora* leaves were screened against *S. aureus*, *E. coli* NCIB 86, *B. subtilis* NCIB 3610, and *P. aeruginosa* NCIB 950 using the agar well-diffusion method with streptomycin as a control. The mean zone of inhibition (ZOI) ranged between 10.1 and 17.1 mm, equivalent to the solvents (hexane, ethyl acetate, and butanol) used in the study (Oloyede et al., 2010), thereby getting negative results. Similar findings were reported (Essiett and Ajibesin, 2010) for antimicrobial evaluation of ethanolic extract of leaves against six human pathogenic bacteria—*S. aureus* NCIB 8588, *B. subtilis* NCIB 3610, *E. coli* NCIB 86, *Proteus vulgaris* (*P. vulgaris*) NCIB 67, *P. aeruginosa* NCIB 950, and *Klebsiella pneumoniae* (*K. pneumoniae*) NCIB 418—and two clinical fungal isolates, *C. albicans* and *A. flavus*. The obtained results revealed that the extract exhibits moderate inhibition against *P. aeruginosa* NCIB 950, *P. vulgaris* NCIB 67, and *A. flavus* with ZOI of 15 ± 3.6 mm, 11 mm, and 9 mm, respectively. The highest MIC value of 250 µg/ml was observed against *P. aeruginosa* NCIB 950 and *A. flavus*. However, no activity was observed with other tested strains (four bacterial and one fungal) (Table 3).

Akinpelu et al. (2015) reported a broad-spectrum antimicrobial activity of hydroalcoholic extract from *A. laxiflora* leaves. The antibacterial and antifungal activity of the *A. laxiflora* extract was evaluated against a panel of bacterial (39) and fungal (17) isolates. The extract at a concentration of 25 mg/ml inhibited all the bacterial isolates, with the ZOI ranging between 12 and 24 mm and MIC ranging between 0.78 and 25 mg/ml. Similarly, at the concentration of 35 mg/ml, the *A. laxiflora* extract inhibited 15 isolates out of 17 fungal isolates, with ZOI ranging between 11 and 23 mm and MIC ranging between 8.75 and 35.00 mg/ml. The highest antibacterial, antifungal activity of *A. laxiflora* was observed against *Shigella* species (24 ± 0.50 mm; MIC 3.13 mg/ml) and *Trichophyton tonsurans (T. tonsurans)* (23 ± 0.50 mm; MIC 2.19 mg/ml), respectively.

Another investigation of the antibacterial activity of *A. laxiflora* was conducted by Mbaveng et al. (2015). The antibacterial activity of a novel flavonoid, 3,4,3′-tri-O-methylellagic acid, isolated from the stem bark of *A. laxiflora* was evaluated against a panel of 14 g negative multi-drug resistant (MDR) bacteria, including strains of *E. coli* (ATCC 8739, AG102, AG100 A_tet_), *Enterobacter aerogenes (E. aerogenes)* (ATCC 13048, CM64, EA27), *K. pneumoniae* (ATCC 11296, KP55), *Providencia stuartii* (*P. stuartii*) (ATCC29916, PS299645), *Enterobacter cloacae* (*E. cloacae*) (BM47, BM67), and *P. aeruginosa* (PA01, PA124) using chloramphenicol as a standard antibiotic. Compound 3,4,3′-tri-O-methylellagic acid exhibited weak antibacterial activity with MIC values ranging from 64 to 256 µg/ml on 4/14 (29%) and more than 256 µg/ml on 8/14 (57%), with no activity on the 2/14 (14%) tested bacteria. The lowest MIC value of 64 µg/ml was obtained against *P. stuartii* ATCC29916. Likewise, the methanolic extract from the leaves and stem bark of *A. laxiflora* was tested for their antibacterial activity against sensitive and resistant strains of bacteria, namely, *P. aeruginosa* (PA01, PA124), *K. pneumoniae* (ATCC11296, KP55, KP63), *E. aerogenes* (ATCC13048, CM64, EA 27, EA 289), *E. coli* (ATCC8739, ATCC10536, AG100ATet, AG102), and *P. stuartii* (ATCC29916, NEA 16) using rapid INT colorimetric assay. The result showed that except for *E. coli* AG100ATet and *P. aeruginosa* PA124, all other test strains (13/15, 86.7%) exerted sensitivity to methanolic leaves and stem bark extract of *A. laxiflora* with a MIC value range of 64–1,024 µg/ml. The *A. laxiflora* bark extract exhibited the highest antibacterial activity with a MIC value of 64 µg/ml against *E. aerogenes* EA 289 (Tchinda et al., 2017).

The different extracts (aqueous, ethyl acetate, and ethanol) of *A. laxiflora* leaves were tested against the clinical strains of *Salmonella typhi* (*S. typhi*) and *Salmonella paratyphi (S. paratyphi)* isolated from human stool. The extract showed dose-dependent inhibition in both the tested strains in different concentrations, likely 20, 40, and 60 mg/ml. However, ethyl acetate extract at the concentration of 60 mg/ml showed the highest antibacterial activity against *S. typhi* and *S. paratyphi* with ZOI of 24 and 19 mm, respectively (Osuntokun and Olajubu, 2015). Similar results were reported in another study by Wansi et al. (2017), in which the extract of methanol leaves exhibited preferred activity against *S. typhi* and *Shigella flexneri* (*S. flexneri*) with MIC values of 512 μg/ml and 1024 μg/ml, respectively.

Osabiya et al. (2017) demonstrated that aqueous and ethanol extracts from the leaves of *A. laxiflora* inhibited six bacterial strains, namely, *B. subtilis*, *S. aureus*, *E. coli*, *Enterococcus faecalis (E. faecalis)*, *K. pneumoniae*, and *S. typhi*. The MIC ranged between 2.5 and 40 mg/ml. However, it was a noteworthy result that the ethanol extract exhibited more potent inhibitory activity at a concentration of 60 mg/ml against *S. aureus* (19.33 ± 0.58 mm) and *K. pneumoniae* (18.33 ± 0.58 mm) compared with the antibiotic chloramphenicol (11.67 ± 0.57 mm and 10.01 ± 0.00 mm). Furthermore, *in vitro* antibacterial activity of essential oils from leaves of *A. laxiflora* was carried out against *B. cereus* ATCC 10872, *B. subtilis* ATCC 6633, *S. aureus* ATCC 25923, *P. aeruginosa* ATCC 9027, and *E. coli* ATCC 25922. The mean ZOI ranged between 7 and 9 mm in contrast to the standard antibiotic ampicillin, with ZOI ranging between 6 and 8 mm (Otuechere et al., 2019).

On account of the traditional use of *A. laxiflora* leave decoction and infusion for the treatment of digestive and gastric disorders, Ngnameko et al. (2019) investigated the anti-*Helicobacter pylori* (*H. pylori*) activity of *A. laxiflora* leave extract. The extract was active against *H. pylori* at a MIC of 20 mg/ml. Lately, *in vitro* antibacterial activity of hexane, chloroform, ethyl acetate, methanol, ethanol, and aqueous extracts of the leaves, roots, and stem bark of *A. laxiflora* on the skin, gastrointestinal, respiratory, and urinary pathogens was also conducted. The extracts were tested against four Gram-positive bacteria, namely, *B. cereus* ATCC 11778, *E. faecalis* ATCC 29212, *S. aureus* ATCC 25923, and *Staphylococcus saprophyticus (S. saprophyticus)* ATCC 15305, and four Gram-negative bacterial strains, namely, *E. coli* ATCC 25922, *K. pneumoniae* ATCC 13883, *Moraxella catarrhalis* (*M. catarrhalis*) ATCC 23246, and *Proteus mirabilis* (*P. mirabilis*) ATCC 43071 using ciprofloxacin as a standard antibacterial agent. All the extracts were effective against most of the tested Gram-positive strains, with MIC ranging between 50 and 63 μg/ml. In addition, bioactivity-guided fractionation of methanolic extract of the *A. laxiflora* stem results in the isolation of ellagic acid, 3-O-methylellagic acid, 3-O-β-D-glucopyranosyl-β-sitosterol, 3-O-acetyl-oleanolic acid, and 3-O-acetyl-ursolic acid. All the compounds displayed antibacterial activity against tested strains with MIC values as low as 4 μg/ml (Siwe-Noundou et al., 2019) (Table 3).

Based on the literature survey, certain gaps were identified in the reported studies. For example, Osabiya et al. (2017) did not mention the strain collection numbers of investigated bacterial strains, which makes it difficult to establish a comparison with other studies. Similarly, limitations were observed in the studies reported by Ngnameko et al. (2019), Osuntokun and Olajubu (2015), and Wansi et al. (2017). For the purpose of quality control, the European Committee on Antimicrobial Susceptibility Testing (EUCAST) has recommended strain collection numbers from the distributors such as ATCC (American Type Culture Collection, United States), NCTC (National Collection of Type Cultures, United Kingdom), CIP (Collection de Institut Pasteur, France), CECT (Coleccion Espanola de Cultivos Tipo, Spain), CCUG (The Culture Collection University of Gothenburg, Sweden), and DSM (Deutsche Stammsammlung fur Mikroorganismen und Zellkulturen, Germany) (Matuschek et al., 2014). Furthermore, the assessment of anti-microbial activity using only the disc diffusion method is a preliminary approach because it did not ascertain the exact concentration-causing antimicrobial effect. Oloyede et al. (2010) reported the ZOI of different extracts determined by the disc diffusion method only. Unfortunately, they did not use agar or broth dilution tests to determine the MIC values. Moreover, the use of the agar diffusion method to determine the antimicrobial activity of plant extracts is considered inadequate owing to the lack of diffusion of non-polar molecules into the aqueous agar matrix, insensitivity, and non-reproducibility of the results in different laboratories. Therefore, serial microplate dilution methods using INT or rezurasin as indicators of growth are the preferred methods to determine realistic and reproducible MIC values (Eloff, 2019). Only a few studies reported the outcomes of rapid INT colorimetric assay. Plant extracts displaying MIC value ≤100 μg/ml are considered to possess noteworthy antimicrobial activity (Bueno, 2012). Nonetheless, multiple studies included in this review have reported MIC values higher than the accepted limit of ≤100 μg/ml. It is often not determined whether the antimicrobial activity is caused by general toxicity to all cells or a selective activity against the microorganisms.

### Anti-amoebic and anti-plasmodial activity

Moundipa et al. (2005) investigated the *in vitro* amoebicidal activity of methanolic leave extract of *A. laxiflora* against clinical isolates of *Entamoeba histolytica* (*E. histolytica*). The extract, at a concentration of 100 μg/ml, showed 60.43% and 52.17% mortality on day 2 and day 4, respectively, indicating anti-amoebic activity.

Okokon et al. (2017b) investigated the *in vitro* antiplasmodial activity of *A. laxiflora* leaves, crude ethanol, petroleum ether, chloroform, ethyl acetate, and butanol extracts against two *Plasmodium falciparum* (*P. falciparum*) strains: CQ sensitive Pf-3D7 and CQ resistant Pf INDO SYBR green assay method. The crude ethanol, petroleum ether, chloroform, ethyl acetate, butanol, and aqueous leave extract of *A. laxiflora* were found to be active *in vitro* with CQ-sensitive IC_50_ values of 31.57 ± 0.94, 27.85 ± 0.36, 26.06 ± 0.19, 9.92 ± 0.28, >100, >100 μg/ml, respectively, and had CQ-resistant IC_50_ values of 16.38 ± 0.94, 23.47 ± 0.15, 14.47 ± 0.35, 7.51 ± 0.24, 52.63 ± 0.22, and >100 μg/ml, respectively. However, the ethyl acetate fraction exhibited the most promising activity against both strains of *P. falciparum*. Further, the fractionation of ethyl acetate extract led to the isolation of 34 phytoconstituents, including polyunsaturated fatty acids (PUFA), phenolics, and flavonoids. Likewise, prophylactic, suppressive, and curative effects of ethanol *A. laxiflora* leave extract (200–600 mg/kg, p. o.) were tested *in vivo* using *Plasmodium berghei* (*P. berghei*) infected mice. The extract showed promising antimalarial activity (Table 4; Figure 5). Similar results were reported by Okokon et al. (2017a) with the *A. laxiflora* root extract. The root extract and fractions also exerted moderate activity against CQ-sensitive (Pf 3D7) and CQ-resistant (Pf INDO) strains of *P. falciparum*, with ethyl acetate fraction exerting the highest activity with an IC_50_ value of 38.44 ± 0.89 and 40.17 ± 0.78 μg/ml in Pf 3D7 and Pf INDO strains, respectively. Recently, an *in vivo* antiplasmodial activity of methanol and chloroform leave extract of *A. laxiflora* was reported (Table 4; Figure 5). Briefly, the extract was administered orally (200–600 mg/kg) to *P. berghei*-infected mice. After 5 days of the treatment study, methanol extract exhibited significantly higher prophylactic, suppressive, and curative activity than the chloroform extract. These studies confirmed the ethnopharmacological use of *A. laxiflora* as a promising indigenous antimalarial drug (Oluyemi and Blessing, 2019) (Table 4).

### Larvicidal activity

Morah and Uduagwu (2017) investigated the larvicidal activity of ethanol and petroleum ether extracts from the leaves of *A. laxiflora* against the third and fourth instar *Anopheles* mosquito larvae. Larva mortality was observed for both petroleum ether and ethanol extract at all concentrations assayed (0.08, 0.1, 0.15, 0.2 mg/ml), with the lowest activity at 0.08 mg/ml and the highest mortality rate observed at 0.2 mg/ml. Moreover, petroleum ether extract exhibited the highest 78% mortality compared to 68%, by ethanol extract at a similar dose of 0.2 mg/ml. In contrast, the larvicidal activity was attributed to the presence of methyl palmitate, icosyl oleate, and diisooctyl phthalate (Table 4; Figure 5).

### Anti-HIV potential

Various extracts of *A. laxiflora* leaves, stem, and roots were evaluated for their Anti-HIV activity using HIV-1 integrase strand transfer assay. HIV-1 integrase inhibitory activity dwelt in most of the root extracts of *A. laxiflora*. The methanolic extract of *A. laxiflora* root exhibited noteworthy HIV-1 integrase inhibitory activity at an IC_50_ value of 0.21 ng/ml compared to chicoric acid taken as a reference (IC_50_ = 6.82 μM) without any significant cytotoxicity against HeLa cells. The ethanolic root extract and ethyl acetate root extract also exhibited marked HIV-1 integrase inhibitory activity with IC_50_ values of 67.07 and 6.034 μg/ml, respectively. Moreover, the inhibitory activity of five compounds (ellagic acid, 3-O-methylellagic acid, 3-O-β-D-glucopyranosyl-β-sitosterol, 3-O-acetyl-oleanolic acid, and 3-O-acetyl-ursolic acid) isolated from the methanolic stem extract of *A. laxiflora* was also investigated on HIV-1 integrase, although all the isolated compounds were non-cytotoxic. However, they did not exhibit significant anti-HIV-1 integrase activity. Only ellagic acid laid out the best HIV-1 integrase inhibitory activity with an IC_50_ value of 90.23 μM. The IC_50_ values of other isolated compounds (3-O-methylellagic acid, 3-O-β-D-glucopyranosyl-β-sitosterol, 3-O-acetyl-oleanolic acid, and 3-O-acetyl-ursolic acid) were either >100 μM or could not be determined (Siwe-Noundou et al., 2019) (Table 4; Figure 5).

### Antidiabetic activity

The use of *A. laxiflora* leave decoction for treating diabetes is common among the Badagry people of Nigeria. This practice was validated through *in vitro* and *in vivo* studies. Ogbole et al. (2016) showed that methanolic extract obtained from *A. laxiflora* leaves inhibits α-amylase moderately with an IC_50_ value of 295.60 μg/ml. The anti-diabetic properties of *A. laxiflora* have recently been studied using an *in vivo* alloxan-induced diabetic rat model. When methanolic extract of *A. laxiflora* leaves was administered orally at a dose of 500 mg/kg, it showed a significant (*p* < 0.05) anti-hyperglycemic effect by lowering blood glycemic levels in diabetic rats (Nimenibo-uadia, 2018) (Table 4).

### Analgesic and anti-inflammatory activity

In order to substantiate the folklore medicinal use of *A. laxiflora* roots infusion or decoction as treatment of malaria and associated pain-related symptoms, Okokon et al. (2017a) investigated the analgesic activity of the crude ethanol extract and other solvent fractions of *A. laxiflora* using mice against the standard drug acetylsalicylic acid (ASA; 100 mg/kg) by acetic acid-induced writhing, hot plate, and formalin-induced hind paw licking methods. The administration of *A. laxiflora* root extract ((75, 150, and 225 mg/kg, p.o.) and extract fractions (150 mg/kg, p.o.) demonstrated a significant (*p* < 0.05–0.001) and dose-dependent analgesic activity in all used pain models. The ethyl acetate fraction exerted the highest analgesic activity compared with the standard drug ASA. The aqueous and methanol extracts from *A. laxiflora* leaves were appraised for analgesic effect in mice*.* The hot plate and tail immersion tests were used to evaluate the central analgesic effects, whereas the acetic acid-induced abdominal writhing assay was used to evaluate peripheral analgesic activity. The extract increased the mean reaction time to pain significantly in the hot plate and tail immersion tests. Additionally, the number of abdominal writhes also decreased substantially, endorsing the folklore use of this plant (Nwonu et al., 2018a).

The anti-inflammatory activity of acetone extract from *A. laxiflora* leaves was investigated *in vitro*. In the study, *A. laxiflora* extracts exerted dose-dependent inhibition of NO production by LPS-activated malignant macrophage cell line RAW264.7 at doses 6.25, 12.5, 25, and 50 μg/ml. At the concentration of 25 μg/ml, the extract exhibited the highest percent inhibition (96.53%). Simultaneously, the *A. laxiflora* extract significantly inhibited 15-LOX activity with an IC_50_ value of 46.03 μg/ml compared to quercetin used as a reference drug (35.85 μg/ml) (Dzoyem and Eloff, 2015). Furthermore, Ngoungoure et al. (2019) reported that methylene chloride/methanol (1:1; v/v) extract obtained from leaves of *A. laxiflora* demonstrated similar results. The *A. laxiflora* extract exerted 68.10% NO inhibition (IC_50_ = 66.57 μg/ml) and 54.58% 15-LOX inhibitory activity (IC_50_ = 90.42 μg/ml) (Table 4).

### Anti-diarrheal activity

To substantiate the ethnomedicinal use of *A. laxiflora* leaves in Cameroon to treat some gastrointestinal disorders, Wansi et al. (2017) conducted an *in vivo* study to appraise the antidiarrheal activity of methanolic and aqueous extracts of *A. laxiflora* (125, 250, and 500 mg/kg) using *S. flexneri*-induced infectious, castor oil-induced secretory, and magnesium sulphate-induced osmotic diarrhea in rats. The methanolic extract showed promising antidiarrheal activity in all animal models. The *A. laxiflora* methanolic extract significantly prolonged the latency period at all doses in the model of osmotic diarrhea, with the 250 mg/kg dose giving the highest 71.6% prolongation in latency time (Table 4).

### Hepatoprotective activity

In the Bamun folk medicine (Cameroon), *A. laxiflora* is reported to treat hepatitis and other liver-related disorders. As toxic hepatitis is often associated with the oxidative destruction of lipids and proteins, Njayou et al. (2008) carried out *ex vivo* rat liver microsomal lipid peroxidation (enzymatic and non-enzymatic) and protein oxidation inhibitory potential of methanol-methylene chloride extract of *A. laxiflora* leaves. On the whole, the *A. laxiflora* extract inhibited the biochemical process in a dose-dependent manner (10, 100, and 200 μg/ml) with the significant inhibition of microsomal lipid peroxidation (95.90 ± 0.57% and 79.17 ± 1.57%; enzymatic and non-enzymatic, respectively) and protein oxidation (95.60 ± 0.59%) at 200 μg/ml concentration.

The ethyl acetate extract of *A. laxiflora* was studied for its possible hepatoprotective effect against CCl_4_-induced hepatotoxicity in rats to rationalize some folklore use. The ethyl acetate extract of *A. laxiflora* at 100 mg/kg body weight significantly counteracted the CCl_4_-induced liver damage by lowering the elevated marker enzymes, namely, ALT, AST, ALP, and LDH levels in the blood to 18.872, 7.054, 22.864, and 180.321, respectively, compared to CCl_4_ elevated levels of 35.712, 12.513, 27.509, and 480.312 for ALT, AST, ALP, and LDH, respectively. Further, histopathological analysis of the liver showed that the ethyl acetate extract may protect the liver from centrilobular necrosis, vacuolization, and macrovesicular fatty change set up by CCl_4._ The hepatoprotective activity was ascribed to four isolated flavonoids, namely, quercetin, quercetin-3,7.3′,4′-tetrasulphate, quercetin-3-O-β-D-glucopyranoside, and quercitrin (Oloyede et al., 2011) (Table 4; Figure 6). In a separate study, *A. laxiflora* was tested for its ability to alleviate sodium arsenate-induced liver damage in Wistar rats. The hexane extract of the *A. laxiflora* leaves could significantly reverse the liver damage caused by sodium arsenate (Esosa et al., 2013). Similarly, the methanolic extract of *A. laxiflora* leaves significantly modulated the levels of liver biochemical parameters GGT, GST, ALT, and AST (Uhunmwangho et al., 2016). The hepatoprotective activity of *A. laxiflora* roots has recently been investigated using sodium arsenate-induced hepatotoxicity as a model. The hexane root extract could significantly lower the liver enzymes (ALT, AST, and ALP), liver metabolizing enzymes (NAD, GST, and Cytochrome b5), and other biochemical parameters (total protein, albumin level, and globulin) (Uhunmwangho et al., 2018). These findings backed up the folklore’s claim that this botanical drug can help with liver problems. (Table 4).

### Anti-anemic activity

Oladiji et al. (2014) investigated the anti-anemic potential of aqueous leave extract of *A. laxiflora* at doses 100, 200, and 300 mg/kg, p.o. on hematological indices (PCV, Hb, RBC, MCV, MCH, and MCHC) of iron-deficient rats. The extract could significantly increase the hematological indices at all doses, compared with the reference drug ferrous sulfate and iron-sufficient rats, attesting to its folklore medicinal use in the treatment of anemia. Similar results were reported by Bada et al. (2017) with an ethanolic extract of *A. laxiflora* leaves. Another study was conducted on the anti-sickle cell anemic activity of *A. laxiflora*. The Anti-sickling activity of methanolic extract from *A. laxiflora* leaves was reported by Bamimore and Elujoba (2018). The extract had 98.8% sickling inhibitory action when given at a level of 8 mg/ml (Table 4; Figure 5).

### Tocolytic and fertility-promoting activity

Traditional African medicine uses the leaf of *A. laxiflora* to treat various reproductive system diseases, including preterm labor, miscarriage, menstrual disorders, postpartum discomfort, fibroids, and infertility. To appraise the ethnomedicinal use of *A. laxiflora* leaves to prevent preterm labor or miscarriage, Bafor et al. (2015) investigated the effect of methanolic extract of *A. laxiflora* leaves on female reproductive structures. Briefly, non-pregnant female mice were orally administered methanolic leave extract (100 and 1,000 mg/kg) for 6 days, using progesterone (10 mg/kg s.c.) as a positive control. The results revealed that the *A. laxiflora* extract at a lower dose (100 mg/kg) exerted progesterone-like activity on the ovaries, uterus, and cervical glands asserting its folklore use in maintaining pregnancy. In another investigation, *ex vivo* uterine contraction modulatory activity of methanolic extract of *A. laxiflora* was conducted using uterine tissue preparation isolated from female albino mice. The extract significantly inhibited spontaneous, oxytocin, and potassium chloride-induced uterine contractions, possibly *via* calcium and potassium ions channel interaction. Furthermore, the authors also isolated three important bio-constituents, namely, 3-deoxy-arabino-hept-2-ulosonic acid, 17-hydroxyingenol, and pheophorbide A, possibly contributing to the methanolic *A. laxiflora* extract activity (Bafor et al., 2018).

Uhunmwangho et al. (2016) studied the effect of the methanolic *A. laxiflora* leave extract on CCl_4_-induced reproductive toxicity. The oral administration of the extract at a graded dose (0.1, 0.5, 1.0, 10.0, and 50 mg/kg body weight) in male Wistar rats for 7 days, significantly reversed the toxic effects of CCl_4_ and aided in male fertility by significantly increasing the percentage motility of sperm and inhibiting sperm morphological aberrations compared with positive control (normal saline) (Table 4).

### Anticonvulsant and sedative activity

The leaves of *A. laxiflora* are traditionally used in the form of decoction or maceration to treat epilepsy and sleeplessness. Hence, the *in vivo* anti-convulsant and sedative activity of *A. laxiflora* was evaluated by Bum et al. (2009). The aqueous extract of the leaves of *A. laxiflora* was investigated for anticonvulsant and sedative activity in male Swiss mice using MES, PTZ, NMDA, INH, PIC, and STR-induced convulsions or turning behavior and diazepam sleep-induced animal models. The aqueous extract at a dose of 60 mg/kg protected 100% of mice against NMDA-induced turning behavior, and at a dose of 120 mg/kg, it protected 75% of mice against STR-induced seizures. The *A. laxiflora* extract failed to provide any significant protection against MES, PTZ, INH, and PIC-induced seizures. Moreover, in the diazepam-induced sleep test, *A. laxiflora* was insignificant in modifying the sleep duration of the control group, indicating the non-sedative activity of *A. laxiflora*. However, a recent study by Nwonu et al. (2018b) demonstrated the anxiolytic and sedative effects of aqueous and methanol extracts of *A. laxiflora* leaves using a staircase climbing test in mice. The results showed that both the methanol and the aqueous extracts of *A. laxiflora* leaves had significant sedative activity at a high dose (800 mg/kg) by significantly decreasing staircase climbing (Table 4).

### Anti-Alzheimer activity

The cholinergic deficit is implicated in the pathogenesis of neurodegenerative disorders such as Alzheimer’s disease and associated progressive memory loss and cognitive function impairments. The cholinergic deficit is the result of a reduction in central nervous system ACh activity due to the AChE-related breakdown of ACh. The extracts from different parts of *A. laxiflora* have been screened for AChE and BuChE inhibitory activity. The first report is that of Dzoyem and Eloff (2015), reporting the AChE inhibition activity of the acetone extract of *A. laxiflora* leaves using the modified Ellman method. The extract exhibited significant but weak inhibitory activity with an IC_50_ value of 364.12 ± 2.39 μg/ml compared to eserine with an IC_50_ value of 4.94 ± 0.015 μg/ml. Similarly, the methylene chloride: methanol (1:1; v/v) extract at a single concentration of 200 μg/ml exhibited 36.02 ± 0.18% AChE inhibitory activity with >200 μg/ml, IC_50_ value compared to galantamine (100 μg/ml; IC_50_ value 24.65 ± 2.12 μg/ml); a standard drug (Ndam Ngoungoure et al., 2019). Elufioye (2017) investigated the effects of various extracts (hexane, ethyl acetate, and aqueous) obtained from *A. laxiflora* leaves, stem bark, and root bark in inhibiting both AChE and BuChE. The results reported that the *A. laxiflora* stem bark and root bark extracts showed selective AChE inhibitory activity with percent inhibition ranging from 10.69% to 34.20% (Table 4).

### Antipsychotic activity

*In vivo* antipsychotic effects of aqueous and methanol extracts of *A. laxiflora* leaves were evaluated by Nwonu et al. (2018c). According to their findings, oral administration of the aqueous and methanol extracts in mice at graded doses (100, 200, 400, 800, and 1,600 mg/kg, p.o.) significantly reduced apomorphine-induced climbing and stereotypic behavior in mice at all tested doses, compared with chlorpromazine, a psycholeptic agent (Table 4).

### Anti-Parkinson’s disease activity

Ngoungoure et al. (2019) assessed whether *A. laxiflora* methylene chloride: methanol (1:1; v/v) leave extract with antioxidant and anti-inflammatory activities could serve as a protective agent against aminochrome-induced toxicity in human astrocytoma cells (U373MG and U373MGsiGT6 cell lines). The results indicated that *A. laxiflora* extracts at doses 0.1 and 1.0 μg/ml significantly altered the aminochrome-induced (75 μM) cell death and mitochondrial membrane potential reduction in both cell lines, implicating the potential usefulness of *A. laxiflora* in Parkinson’s disease. However, further studies are warranted in isolating the active constituents and detailed elucidation of the mechanism of the action for safe and effective utility in Parkinson’s disease (Table 4).

### Anxiolytic activity

The anti-anxiety efficacy of aqueous and methanol extracts of *A. laxiflora* in mice was tested using the elevated plus-maze and the staircase behavioral paradigms to indicate the ethnomedicinal usage of *A. laxiflora* as an anxiolytic drug (Nwonu et al., 2018b). *A. laxiflora* exerted a significant anxiolytic effect on the elevated plus maze and staircase animal model against the standard drug diazepam (0.1 mg/kg, p.o.). Albino mice were divided into seven groups of six animals each. The control group (I) received 10% Tween-80 (10 ml/kg, p.o.), whereas the test groups (II–VI) received *A. laxiflora* aqueous or methanol leave extract in graded doses (100, 200, 400, 800, 1,600 mg/kg, p.o.) and standard drug group (VII) received standard drug diazepam (1 mg/kg i.p.) 30 or 60 min before the experiments. In the elevated plus maze model, the *A. laxiflora* methanol extract significantly increased the percent entry and percent time spent in open arms at lower and higher doses (100, 200, 800, and 1,600 mg/kg). In contrast, almost all doses significantly decreased the index of open-arm avoidance, attesting to anxiolytic activity. Similar results were observed for the aqueous extract of *A. laxiflora* leaves at doses 400 and 1,600 mg/kg, p.o., validating its anti-anxiety effect. In the staircase paradigm also, the methanol and the aqueous extracts of *A. laxiflora* leaves exhibited anti-anxiety activity by decreasing staircase rearing (aqueous extract: all doses; methanol extract: 400, 800, and 1,600 mg/kg, p.o.) and increasing staircase step-climbing (aqueous extract: 200 and 800 mg/kg p.o.; methanol extract: 100 and 200 mg/kg, p.o.) behaviors significantly (Table 4).

### Antioxidant activity

In the Ugba region of Nigeria, the leaves of *A. laxiflora* are traditionally used to wrap food items for preservation. Farombi et al. (2003) were the first to report the antioxidant activity of hexane and methanol extracts from *A. laxiflora* leaves and roots using the ferric thiocyanate method, horseradish peroxidase catalyzed oxidation of ABTS, β-carotene linoleate model system, and rat liver microsomal lipid peroxidation assay. The antioxidant activity was observed in the following order: hexane root extract (76.4%) > methanol root extract (63%) > methanol leave extract (40%) > hexane leave extract (38%) at 0.05% concentration. The hexane root extract’s antioxidant activity was compared with that of BHA (80%), a standard antioxidant. Another report indicated the antioxidant activity of hydroethanolic extract and solvent fractions of *A. laxiflora* leaves by the DPPH spectrophotometric assay method. All the test samples showed less scavenging activity (EC_50_ 12.97, 24.34, and 106.74 μg/ml for ethyl acetate, n-butanol, and crude ethanol extract, respectively) relative to the reference standard ascorbic acid (EC_50_ 4.78 μg/ml). Moreover, bioassay-guided fractionation of n-butanol fraction led to the isolation of two flavonoids, namely, taxifolin glycoside and quercitrin, suggesting their involvement in observed antioxidant activity (Adeloye et al., 2005).

According to Oloyede et al. (2010), the H_2_O_2_ scavenging activity of the butanol extract of *A. laxiflora* through the FTC method was compared favorably with standard reference α-tocopherol at 500 μg/ml concentration. The scavenging activity was observed for the various extracts (hexane, ethyl acetate, butanol, and aqueous) of leaves at various concentrations (50, 100, 250, and 500 μg/ml) assayed in a concentration dependant manner. Similarly, the acetone leave extract showed significant (*p* < 0.05) antioxidant activity in DPPH, ABTS, and FRAP assays with IC_50_ values of 17.19 ± 1.02, 18.53 ± 1.42, and 438.42 ± 15.55 μg/ml, respectively. Trolox was used as a standard antioxidant with IC_50_ values of 3.14 ± 0.10 and 6.05 ± 0.24 μg/ml in DPPH and ABTS assay, respectively (Dzoyem and Eloff, 2015). The *in vivo* antioxidant assay of methanolic extract was investigated by determining the effects on serum CAT, SOD, and GSH enzymes in experimental animals. The extract at different concentrations (0.5, 1.0, and10.50 mg/kg body weight, p.o.) significantly (*p* < 0.05) raised the GSH, SOD, and CAT activity (Uhunmwangho et al., 2017). Lately, Morah and Uduagwu (2017) assessed the radical scavenging activity of petroleum ether and ethanol extract of *A. laxiflora* using a DPPH spectrophotometric assay. Both extracts at all concentrations (40, 80, 100, 150, and 200 μg/ml) exhibited a dose-dependent anti-DPPH activity. The petroleum ether and ethanol extract at 200 μg/ml concentration exhibited greater percent DPPH radical scavenging ability (50.50% and 42.95%, respectively) than ascorbic acid at the same concentration (35.22%). The antioxidant activity of extracts was attributed to the isolated compounds, namely, 3-acetoxy-7,8-epoxylanostan-11-one, rhodopin, ethyl iso-allocholate, hexadecanoic acid, 9-octadecenyl hexanoate, eicosyl oleate, and astaxanthin. The results laid the credence for the ethnobotanical food preservative and natural oxidant use of *A. laxiflora* (Table 4).

### Anti-cancer activity

Although *A. laxiflora* plant parts are traditionally used as an alternative medicine for cancer treatment, there is a paucity of experimental and clinical data on the anticancer activity of *A. laxiflora* (Dzoyem and Eloff, 2015). In the brine shrimp bioassay against *Artemia salina*, the aqueous, ethanol, and methanol extract of *A. laxiflora* was found toxic with LC_50_ values of 41.01, 8.91, and 142.40 μg/ml, respectively, indicating potential anti-cancer properties (Ogbole et al., 2016; Osabiya et al., 2017) (Figure 5). The methanolic extract of *A. laxiflora* root, stem, and leaves had significant anticancer activity against drug-sensitive leukemia CCRF-CEM cell line with IC_50_ values of >80, 49.21 ± 11.16, and 43.67 ± 4.06 μg/ml, respectively (Kuete et al., 2016). In two separate studies, Okokon et al. (2017a, 2017b) investigated the cytotoxicity activity of ethanol extract and solvent fractions (petroleum ether, dichloromethane, ethyl acetate, butanol, and aqueous) of *A. laxiflora* leaves and root against HeLa and HEK cell lines using MTT assay. The *A. laxiflora* root crude ethanolic extracts and fractions were non-toxic in the HeLa cell line with an IC_50_ value greater than 100 μg/ml, whereas the *A. laxiflora* root extracts and fractions were cytotoxic to the tested HeLa and HEK cell lines with potency order of ethyl acetate > chloroform > petroleum ether > crude extract. The ethyl acetate fraction was highly toxic to both cell lines with TC_50_ values of 8.83 and 1.41 μg/ml, respectively, suggesting ethyl acetate fraction could be a potential source of anticancer agents. In contrast, Siwe-Noundou et al. (2019) reported that various extracts (hexane, chloroform, ethyl acetate, methanol, ethanol, and aqueous, 25 μg/ml) of *A. laxiflora* root, stem, and leaves and five isolated compounds from methanolic *A. laxiflora* stem bark, namely, ellagic acid, 3-O-methylellagic acid, 3-O-β-D-glucopyranosyl-β-sitosterol, 3-O-acetyl-oleanolic acid, and 3-O-acetyl-ursolic acid (20 μM), were non-toxic against HeLa cell lines (cell viability >100%). Sandjo et al. (2011) tested the cytotoxicity of six isolated compounds, namely, (10*Z*)-tetradec-10-enoic acid-(2*S*)-2-carboxy-2-hydroxyethyl ester; (2*R*)-2-hydroxy-*N*-[(2*S*,3*S*,4*R*,15*Z*)-1,3,4-trihydroxy-15-triaconten-2-yl]octacosamide; 3-acetyloleanolic acid; 3-acetoxyursolic acid; 3-O-methylellagic acid; and 3-O-methylellagic acid-3′-O-α-rhamnopyranoside, against the HL60 cell line. Compounds 3-acetyloleanolic acid and 3-acetoxyursolic acid exhibited potent cytotoxic effects with IC_50_ values of 6.6 and 6.8 μM, respectively (Table 4).

Though multiple studies have focused on the pharmacological role of *A. laxiflora*, some parts of *A. laxiflora*, such as stem branchlets, fruits, and seeds, have not yet been studied. Indeed, fruits of this plant are used in traditional medicine for curing infertility and fibroid treatment in women. Hence, it is highly recommended to conduct further research in this domain. Moreover, most of the research focuses on *in vitro* pharmacology of *A. laxiflora*. Thus, researchers are strongly recommended to conduct *in vivo* pharmacological and clinical studies to understand the molecular mechanism of action of this botanical drug.

## Toxicity studies

Only a few researchers have evaluated the toxicity of *A. laxiflora*. Farombi et al. (2003) investigated the acute toxicity of the hexane and methanol extract of *A. laxiflora* roots and leaves by administering it in Wistar rats by oral route (100–5,000 mg/kg). After 14 days of monitoring, the extract did not affect mortality. In rats given the drug orally, the LD_50_ was greater than 5,000 mg/kg.

The protective effects of hexane leaf extract of *A. laxiflora* against sodium arsenate-induced (2 mg/kg, p.o.) liver toxicity in Wistar rats was investigated by Esosa et al. (2013). The rats were treated with sodium arsenate for 2 days and suffered elevation in serum and liver biochemical indices (ALT, AST, ALP, GGP, and TB). Pretreatment with the extract of *A. laxiflora* hexane leaves at 10 mg/kg as prophylaxis significantly reversed these changes. Though, post-treatment of animals with the extract after a sodium arsenate treatment did not completely normalize the biochemical indices, suggesting that *A. laxiflora* extract may protect against sodium arsenate-induced hepatic toxicity.

The toxicity of the methanol extract of *A. laxiflora* leaves was evaluated by brine shrimp lethality bioassay. The methanolic extract displayed significant lethality with an LC_50_ value of 142.40 μg/ml (Ogbole et al., 2016). In the same line, Osabiya et al. (2017) reported the high toxicity potential of ethanol and aqueous leaf extracts of *A. laxiflora* by brine shrimp lethality bioassay. The aqueous and ethanol extracts showed strong toxicity with LC_50_ values of 41.01 and 8.91 μg/ml, respectively (Figure 5).

In two separate studies, Okokon et al. (2017a, 2017b) examined the possible development of toxicity caused by ethanol extracts of *A. laxiflora* leaves and roots by administering various doses of extracts intraperitoneally in albino mice. The LD_50_ values were 2,236 and 748.33 mg/kg for leaves and root extract, respectively, indicating that the ethanolic extract might be toxic at higher doses when administered intraperitoneally.

The acute toxicity of the *A. laxiflora* aqueous and methanolic leave extract was assessed using albino mice. In this study, the aqueous extract was well tolerated by the animals up to 1,600 mg/kg (oral, intraperitoneal), whereas the methanol extract was safe up to 400 mg/kg and 1,600 mg/kg, intraperitoneally and orally, respectively (Nwonu et al., 2018b; 2018c; 2018a).

In a 21-day study, the effect of ethanolic extract from *A. laxiflora* was assayed on hematological indices and organ body weight of Wistar rats. The extract had an anemia-ameliorative effect by increasing the RBC, WBC, platelets, PCV, and Hb levels. However, there was an increase in the heart and lung weights at higher doses (200 and 300 mg/kg, p.o.), showing that the plant should be used cautiously when used orally at higher doses for the treatment of anemia (Bada et al., 2017).

Essential oils from *A. laxiflora* leaves obtained by hydro-distillation were also assessed for toxic effects at doses 100, 200, and 400 mg/kg on albino rats. Oral administration of essential oils at higher doses (200 and 400 mg/kg) elevated serum ALT activity, depletion of serum bilirubin, and liver hypertrophy, suggesting possible derangement of hepatic functions at higher doses (Otuechere et al., 2019).

A few reports have highlighted the toxicological aspects of *A. laxiflora*. Although *in vitro* and *in vivo* models have been utilized, only acute effects have been studied. Sub-acute, chronic, mutagenic, and teratogenic effects need to be assessed to support the safe use of this plant. Moreover, the toxicity analysis of chemical constituents is recommended to be carried out rather than its crude extract.

## Clinical studies

Chewing *A. laxiflora* sticks are common oral cleaning practices in Nigeria (Odugbemi and Akinsulire, 2008), considered a good source of fluoride, an essential element for preventing dental caries. To rationalize *A. laxiflora* use as chewing sticks as a viable alternative in providing the required fluoride in poor communities, Emeke et al. (2019) determined and compared the salivary fluoride retention after the *A. laxiflora* stick used with a non-herbal fluorinated product by using a double blind cross over experimental study with 20 participants. Salivary fluoride concentration was determined after 0, 10, 30, 45, and 60 min after chewing stick use. In the results, the baseline salivary fluoride concentration was 25.95 ± 4.58 ppm, whereas after *A. laxiflora* use, the salivary fluoride concentration was 228.0 ± 032.80 ppm. The difference in mean salivary fluoride concentration was statistically significant (*p* < 0.001), indicating that chewing sticks are a cost-effective and efficient means of caries prevention. Although multiple pharmacological effects are assigned to *A. laxiflora*, a smaller number of clinical trials have been conducted. Hence, more clinical studies are required to establish the pharmacological significance or clinical applications of *A. laxiflora.*

## Other applications

The ethanol extract of *A. laxiflora* leaves was evaluated against a field to store pests of maize grains, namely, maize weevil (*Sitophilus zeamais*). The extract displayed significant insecticidal activity in stored maize seeds, suggesting an eco-friendly alternative to synthetic pesticides (Ileke et al., 2020) (Figure 5). The *A. laxiflora* leave extract has been reported to prevent the corrosion of mild steel in an acidic medium (Olasehinde et al., 2015). The *A. laxiflora* stem bark and leave extract has been used as a reducing and capping agent for synthesizing platinum and bimetallic platinum-copper nanoparticles for catalytic oxidative desulfurization of model oil (Olajire et al., 2018; 2017b; 2017a). Similarly, Ekennia et al. (2021) biosynthesized quasi-hexagonal zinc oxide nanoparticles using the aqueous leave extract. Moreover, the synthesized nanoparticles exhibited good photocatalytic activity against Congo red dye solution and mushroom tyrosinase inhibitory activity.

## Conclusion

This review highlights crucial information about the traditional use, phytochemistry, and pharmacological activities of crude extracts, as well as the phytochemicals of *A. laxiflora.* Diverse ethnomedicinal uses are linked with various parts of *A. laxiflora*, including the treatment of malaria, diabetes, sickle cell anemia, inflammatory conditions, skin disorders, and venereal diseases; gastrointestinal problems such as hepatitis, stomachache, dysentery, and piles; and neurological disorders such as anxiety, depression, insomnia, and epilepsy. Although several pharmacological activities of *A laxiflora* have been reported based on ethnomedicine uses, some must be validated pharmacologically. Meanwhile, most pharmacological activities have been reported using crude extracts rather than isolated compounds. As a result, further research is required to determine the links between isolated chemicals and medicinal uses. Phytochemical investigations revealed the presence of alkaloids, flavonoids, terpenoids, phenolic compounds, and fatty acids. The compounds 3-acetyloleanolic acid, 3-acetoxyursolic acid, ellagic acid, and quercetin and their derivatives could be drug candidates for treating HIV, cancer, and microbial infections because of their potent biological activities. This study suggests that in-depth investigations on the mechanism of action, the pharmacological activity of isolated compounds, and toxicological analysis of biologically active extracts and active compounds of *A. laxiflora* are all worthwhile endeavors. Research in these areas could support possible medicinal uses and future development into therapeutic modalities.

## Data Availability

The raw data supporting the conclusion of this article will be made available by the authors without undue reservation.

## Glossary

**AGS**: Accelerated gradient chromatography
**MS**: Mass spectrometry
**NMR**: Nuclear magnetic resonance
**SLHC**: Sephadex-20 column chromatography
**HR-ESI-TOF-MS**: High resolution-electrospray ionization-time of flight-mass spectrometry
**GC-MS**: Gas chromatography-mass spectrometry
**ZOI**: Zones of inhibition
**INT**: Iodonitrotetrazolium
**p.o.**: per oral
s.c.: Subcutaneous
**MES**: Maximal electroshock
**PTZ**: Pentylenetetrazol
**NMDA**: N-Methyl-D-aspartate
**INH**: Isonicotinic hydrazide acid
**PIC**: Picrotoxin
**STR**: Strychnine
**HIV**: Human immunodeficiency virus
**HeLa cells**: human cervix adenocarcinoma
**IC_50_**: Half maximal inhibitory concentration
**ASA**: Amino salicylic acid
**15-LOX**: 15-Lipoxygenase
**NO**: Nitric oxide
**LPS**: Bacterial lipopolysaccharides
**MIC**: Minimum inhibitory concentration
**MBC**: Minimum bactericidal concentration
**CQ**: Chloroquine
**CCl_4_**: Carbon tetrachloride
**ALT**: Alanine aminotransferase
**AST**: Aspartate aminotransferase
**ALP**: Alkaline phosphatase
**LDH**: Lactate dehydrogenase
**GGT**: Gamma-glutamyl transferase
**GST**: Glutathione-s-transferase
**NAD**: 4-Nitroanisole demethylase
**PCV**: Packed cell volume
**Hb**: Hemoglobin
**RBC**: Red blood cell
**MCV**: Mean corpuscular volume
**MCH**: Mean corpuscular hemoglobin
**MCHC**: Mean corpuscular hemoglobin concentration
**ACh**: Acetylcholine
**AChE**: Acetylcholine esterase
**BuChE**: Butylcholine esterase
**ABTS**: 2,2 Azinobis-(3-ethylbenzothiazoline-6-sulfonic acid)
**BHA**: Butylated hydroxyanisole
**DPPH**: 2, 2-Diphenyl-1-picrylhydrazyl
**FTC**: Ferric thiocyanate
**FRAP**: Ferric reducing antioxidant power
**CAT**: Catalase
**SOD**: Superoxide dismutase
**GSH**: Reduced glutathione
**EC_50_**: Half maximal effective concentration
**CCRF-CEM**: Human T lymphoblast cell line
**HEK**: Human embryonic kidney cells
**HL60**: Human promyelocytic leukemia
**MTT**: 3-(4,5-Dimethylthiazol-2-yl)-2,5-diphenyl tetrazolium bromide
**TC_50_**: Median toxic concentration
**LD_50_**: Median lethal dose
**KCl**: Potassium chloride
