# Supplementary material for: Appraising the therapeutical potentials of Alchornea laxiflora (Benth.) Pax & K. Hoffm., an underexplored medicinal herb: A systematic review
Source: Front Pharmacol. 2022 Dec 2;13:958453. doi: 10.3389/fphar.2022.958453 (PMC9761395; doi:10.3389/fphar.2022.958453)
Supplement: Supplementary file 1 [file DataSheet1.docx]

**Supplementary Information**

**4**

5

12

8

7

6

**1**

3

**2**

11

**9; R= Galactopyranoside**

**10; R= Xylopyranoside**

13

**Fig.S1. Chemical structures of isolated flavonoids from *A. laxiflora***

17

16

15

14

197

20

21

22

28

30

32

29

27

26

24

23

25

31

18

**Fig.S2. Chemical Structures of isolated Phenolic compounds from *A. laxiflora***

49

46

45

43

54

53

38

36

34

39

50

48

47

44

42

41

35

33

52

39

51

40

**Fig.S3. Chemical structures of isolated Terpenoids from *A. laxiflora***

**Fig.S4. Chemical structures of isolated Fatty acids from *A. laxiflora***

61

60

57

59

58

55

71

56

73

94

44

72

87

92

97

95

91

90

89

88

69

84

63

76

96

75

83

86

85

81

74

62

93

64

66

67

65

68

77

82

80

70

78

79

**Fig.S5. Chemical structures of isolated Fatty acids from *A. laxiflora***

100

99

98

103

102

101

**Fig.S6. Chemical structures of isolated Alkaloids from *A. laxiflora***

114

107

106

105

104

112

108

115

109

111

113

116

110

119

118

117

120

124

125

121

129

123

122

131

130

126

127

132

128

**Fig.S7. Chemical structures of miscellaneous compounds from *A. laxiflora***
